# Topological stratification of continuous genetic variation in large biobanks

**DOI:** 10.1371/journal.pgen.1012068

**Published:** 2026-03-16

**Authors:** Alex Diaz-Papkovich, Shadi Zabad, Hannah Snell, Chief Ben-Eghan, Luke Anderson-Trocmé, Georgette Femerling, Vikram Nathan, Jenisha Patel, Simon Gravel

**Affiliations:** 1 Quantitative Life Sciences, McGill University, Montreal, Canada; 2 Department of Human Genetics, McGill University, Montreal, Canada; 3 School of Computer Science, McGill University, Montreal, Canada; 4 Center for Computational Molecular Biology, Brown University, Providence, Rhode Island, United States of America; 5 Department of Bioengineering, McGill University, Montreal, Canada; University of Melbourne, AUSTRALIA

## Abstract

Biobanks now contain genetic data from millions of individuals. Dimensionality reduction, visualization and clustering are standard when exploring data at these scales; while efficient and tractable methods exist for the first two, clustering remains challenging because of the many ways in which demography and sampling can affect structure. In practice, clustering is commonly performed by drawing shapes around dimensionally reduced data or assuming populations have “type” genomes or allele frequencies that represent a population. We propose to use dimensionality reduction with UMAP followed by clustering with HDBSCAN to identify sets of points forming relatively dense subsets in genotype space. The approach is fast, easy to implement, and integrates with existing pipelines. When applied to simulated data or data from three biobanks, the approach identifies groups of individuals enriched for shared features correlated with ancestry, including country of birth, ethnicity, and sampling location, without requiring strong assumptions about the number or size of clusters, or the sources of population structure. Because it does not rely on proximity to a specific point in genetic space, this topological approach can form clusters that continuously span long distances in genetic space. This can help distinguish admixed populations, which can exhibit wide ancestry variation within populations and overlap of ancestry proportions across populations. Such clusters can highlight and account for interpretable sources of genetic, demographic, or sampling heterogeneity in a dataset that would otherwise have required a range of specialized techniques. We illustrate how topological genetic strata can further help us understand structure within biobanks, evaluate distributions of genotypic and phenotypic data, examine polygenic score transferability, identify potential influential alleles, and perform quality control.

## Author summary

Clustering is a common approach to study large-scale genomic datasets. It can be used to investigate the relationships between genetics, demography, and biomedical/environmental variables, as well as defining subgroups for downstream study. Identifying clusters in large biobanks present challenges due to their large size, high dimensionality, and the complexity of the demographic and sampling processes that shape observed genetic diversity. We present a computationally tractable method of clustering using dimensionality reduction (UMAP) and density clustering (HDBSCAN) that captures relatedness patterns and reflects important characteristics of a biobank’s data, even where there are subpopulations of widely varying sizes. This method does not depend on external reference panels, and the clusters can be characterized *post hoc* using ancestry and ancestry-associated data without using reductive labels. We carry out clustering on simulated data as well as three biobanks and present a series of vignettes showing how clustering helped us identify patterns due to demographic histories and sampling strategy, with an impact on phenotype distributions, genetic risk score accuracy, and data quality control.

## Introduction

Following improvements in genomic technologies, large-scale biobanks have become commonplace. The Global Biobank Meta-analysis Initiative (GBMI), for example, lists 23 biobanks with genetic data and health records from over 2.2 million individuals [1]. The growth in sample sizes has led to increased potential for scientific findings, with thousands of genetic loci implicated with phenotypes in genome-wide association studies (GWAS), and used to predict disease traits via polygenic scores (PGS). Though the growth of biobanks has fuelled discovery, population structure—excess relatedness within subgroups relative to panmixia—remains a persistent confounder in GWAS and PGS (e.g., [2,3]). Many methods in population genetics seek to describe and account for population structure, but the complexity of human history and of biobank recruitment strategies preclude simple model-based approaches from effectively capturing the many determinants of observed genetic variation.

As an alternative, dimensional reduction and visualization are commonly used to examine both discrete and continuous aspects of genetic variation (e.g., [4,5]). Within the framework of exploratory-confirmatory data analysis, visualization of complex data enables pattern-recognition and the generation and testing of hypotheses [6]. Visualization alone, however, cannot be used for analysis, and visualization techniques are often used as a precursor to stratification. For example, principal component analysis (PCA) can be used to visualize data and individuals within a certain area are commonly deemed to share an ancestry label. Visualizing principal components (PCs) beyond the first two is often done by numerous pairwise comparisons, making visual identification of clusters difficult.

Existing clustering methods based on leading PCs face a conundrum: using too few PCs will exclude relevant population structure and thus not identify some populations; using too many will result in clusters being difficult to identify, particularly for populations without reference panels, complex admixture histories, or small sample sizes [7]. Other approaches create clusters based on shared identity-by-descent (IBD) segments or recent genetic relatedness (e.g., [8,9]). Self-declared variables like race and ethnicity are also sometimes used for genetic stratification but are imperfect indicators of genetic ancestry and are best avoided as proxies for it [10,11]. Finally, in recently admixed populations (i.e., populations who derive ancestry from “source” populations who had been in relative isolation), grouping based on inferred admixture proportions is also common, often with the use of contemporary populations as a proxy for the historic source populations. While not formally a clustering technique, since it does not assign a label to each individual, the approach implies the existence of “source populations” as discrete explanatory variables.

Despite the demand, there is not an effective, fast, and tractable method for stratifying biobank data based on patterns of genetic structure. In practice, researchers often manually group participants into discrete ad hoc “clusters” that they perceive in low-dimensional visualizations, which they use as strata in downstream analyses regarding, e.g., heterogeneity in ancestry and allele frequencies [12], environmental exposures [4], or assessing the performance of PGS [2,13]. There are many drawbacks to such ad hoc approaches. For example, in cosmopolitan cohorts, there can be many subgroups with distinct ancestral histories, leading researchers to manually distinguish between a “majority” cluster and an “everybody else” cluster—often to be discarded [14,15].

We propose a topological data analysis approach as an alternative. Rather than grouping individuals by proximity to a “population centre”, as many clustering approaches do, a topological approach describes the network of neighbourhoods between data points—here, this would be the network of genetic similarity between individuals. It is well-suited to describe collections of points in high-dimensional space with smooth distributions but with no clear centre or “archetype”. We use UMAP, which assumes that the topology of high-dimensional data can be represented with a neighbourhood graph, and can be locally approximated and reconstructed in a low-dimensional space. After reconstructing data in the low-dimensional space, we identify clusters in the data—i.e., the genetic strata—using density clustering. This approach is unsupervised, requiring neither a number of clusters nor a reference panel, and thus fits naturally with population genetic data, which is sparse and contains numerous sub-populations of unknown and varying sizes, often without *a priori* definitions. It is a useful complementary or alternative tool for existing methods, such as ancestral component modelling, for the description of genetic variation in complex cohorts.

Clustering of human genetic variation comes with very concrete risks—a high-profile UMAP-based visualization of the All of Us biobank that highlighted a correlation between race/ethnicity and visible genetic clusters came under intense scrutiny for over-emphasizing this correlation [16]. Others have argued against clustering human genetic data altogether as it can obscure individual-level details in, e.g., PGS estimation [7]. We therefore include a number of vignettes illustrating how topological clustering of genetic data has helped us interpret biobank data beyond broad ancestral categories.

We demonstrate the effectiveness of this approach on three biobanks, showing that we can consistently and effectively identify and characterize sources of population structure in each cohort, and relate many key variables to this structure. We simultaneously identify structured groups as small as 100 individuals and as large as 400,000 within the same cohort in a matter of seconds, and describe environmental, sociodemographic, and phenotype variation across groups. We use stratification to identify populations for which PCA adjustment fails within a biobank (often admixed populations) and populations for which PGS transferability is poor (often, but not always, populations diverged from the training population). Finally, we highlight the role of topological modelling in quality control, a critical aspect biobank analyses.

## Method overview

We propose to perform clustering based on distances computed in genotype space, that is, where each individual is represented by a vector of allele counts for genetic variants. We will use Hierarchical Density-Based Spatial Clustering of Applications with Noise (HDBSCAN) for clustering. This is a general-purpose clustering approach based on building a minimal spanning tree through all points, and then cutting this tree at select branches to generate clusters. Directly building a minimal spanning tree in the original space is sensitive to noise, however. HDBSCAN first computes an *mutual reachability distance*, which accounts for the fact that points that share many neighbours should be considered closer than points that do not, and implements more advanced strategies (cluster stability analysis) to decide which branch to cut.

**Fig 1:**
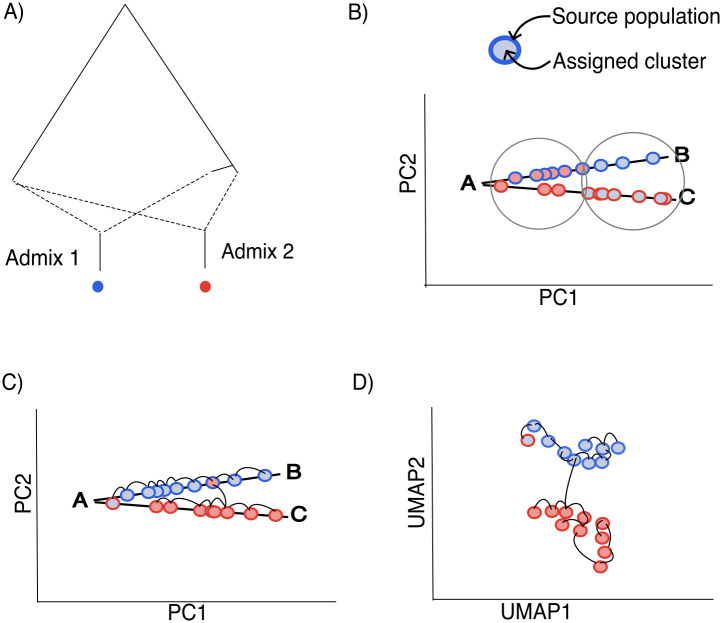
A cartoon of clustering approaches on two admixed populations. The dots represent individuals; their border colours are their source populations, and their internal colours are their assigned clusters. **A)** A schematic of two admixed populations with their source populations. **B)** The circles represent the typological clusters (such as k-means) that identify centres for each cluster, grouping individuals by ancestry proportion from the closely-related source populations. **C)** A minimum spanning tree can rapidly identify paths of genetically similar individuals, allowing for spatially extended clusters. However, it is subject to noise. **D)** Low-dimensional UMAP representations of high-dimensional data are designed to preserve high-dimensional neighbourhoods,and are used to assign clusters. Both typological and topological approaches are oversimplifications of the underlying model and are informative about different aspects of the data.

A motivation for using a topological clustering approach such as HDBSCAN, relative to a typological approach such as k-means, is that populations are not always distributed around a central point. For example, admixed populations are often seen on PCA space as occupying lines or planes (Fig 1). By considering paths in a modified genotype space, a topological approach uses the continuity of points to identify continuously distributed groups of points. As such, topological approaches are also sensitive to sampling schemes that leave gaps in genotype space.

To reduce computational burden, we can also perform analyses on genotype data previously projected to any number of PCs. HDBSCAN has been used before on population genetic data directly on PC-reduced data [17,18]. As we will see, this results in large proportions of individuals discarded as noise.

We explore two modifications to this basic approach: first, a recent implementation of HDBSCAN by Malzer and Baum (HDBSCAN($\hat{\epsilon }$), [19]), which drastically reduces the number of discarded points by adaptively estimating a local density parameter bounded by $1/\hat{\epsilon }$. Second, we preprocess the data using uniform manifold approximation and projection (UMAP) [20], a nonlinear dimensionality reduction method which seeks to preserve the local neighbourhoods from the original space without seeking to preserve distances. This preprocessing has been used in many applications and has been argued to capture high-dimensional neighbourhoods better than the accessibility distance approach alone [21,22].

**Fig 2:**
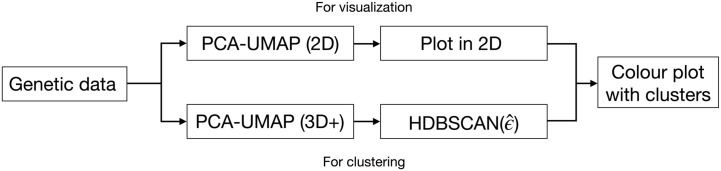
Distinguishing two types of dimension reduction used in this work. We use UMAP for dimension reduction for both visualization and clustering, but the two uses are independent and have different parameter requirements. For visualization, we use PCA-UMAP to reduce data to two dimensions and use a larger minimum distance to improve legibility. While necessary for visualization, reduction to two dimensions leaves little freedom to respect the original topology. Clustering benefits from UMAP reduction to three or more dimensions to strike a balance between preservation of the original data topology and high point density for clustering, and the minimal neighbour distance. The clustering does not depend on the visualization technique, and any visualization method can be used.

UMAP requires three parameter inputs: the target number of dimensions, the number of nearest neighbours (used to define the size of high-dimensional neighbourhoods to approximate), and the minimum distance between pairs of points in the low-dimensional space. We have previously explored its use for visualization in 2 and 3 dimensions [4]. In this work, we will use UMAP both for visualizing data and for preprocessing data for clustering. Both tasks require different parameter choices (Fig 2):

1. For visualization, we reduce data to 2 dimensions and use a relatively high minimum distance (0.3 to 0.5) for visual clarity. Using UMAP for visualization is optional—cluster labels may be paired with any scatterplot (e.g., PCA).
2. For clustering, we reduce data to 3 or more dimensions and use a very low minimum distance between neighbouring points (near or equal to 0) to facilitate algorithmic identification of dense clumps of data.

After reducing genetic data to 3 or more dimensions with UMAP in step 2, we use HDBSCAN($\hat{\epsilon }$) to extract clusters.

In HDBSCAN($\hat{\epsilon }$), users define a minimum number of points required for a cluster to form; the number of clusters is not specified, and the sizes of the clusters are not specified *a priori* and only required to be above the minimum size. The latter two properties are useful to describe biobank data, since they often contain population structure at many scales, and it is usually difficult to specify in advance a useful number of subgroups to consider.

The parameter $\hat{\epsilon }$ controls the local density threshold for cluster splits and merges: low values require clusters to have higher point densities to be considered stable. Setting $\hat{\epsilon }=0$ results in the default HDBSCAN implementation, which (in biobanks) tends to generate uninformative microclusters and classifies many points as noise; higher values of $\hat{\epsilon }$ allow clusters to merge with one another and consequently drastically reduce the number of noise points. In practice we find $0.3\leq \hat{\epsilon }\leq 0.5$ to be useful range for each biobank we studied. We provide more details on parameters in S1 Text.

**Fig 3:**
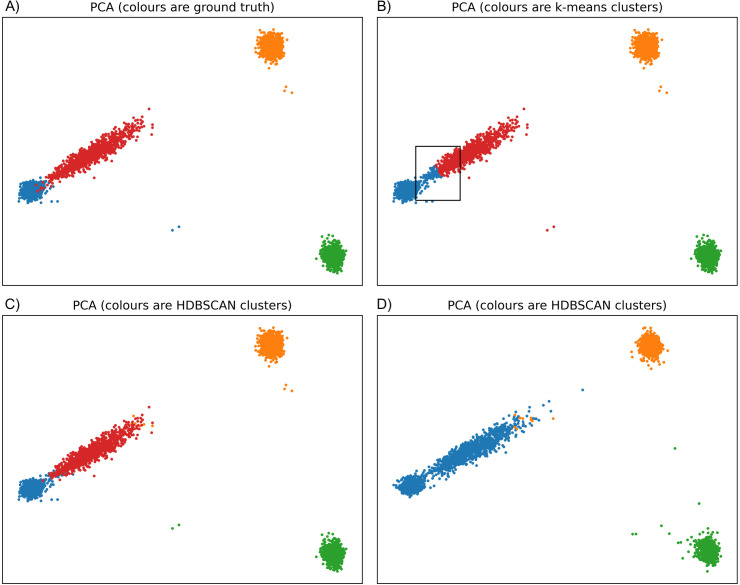
Comparing UMAP-HDBSCAN($\hat{\epsilon }$) to k-means on simulated data. Four simulated populations of 1000 genotypes each: two source populations, one admixed population (75%/25% from each source population) and a fourth population. Each panel presents the top two PCs. **(a)** Colours are population labels (considered ground truth here, though different ground truths could be considered). **(b)** Colours are clusters derived from k-means (*K* = 4) on the top 4 PCs. The box highlights genotypes from the admixed population being clustered with a source population. **(c)** Colours are clusters from UMAP-HDBSCAN($\hat{\epsilon }$); four clusters are extracted and largely match the ground truth. **(d)** Colours are clusters from UMAP-HDBSCAN($\hat{\epsilon }$) from a different replicate. The admixed cluster and a source cluster are merged.

To illustrate how our method contrasts with k-means, we simulated four populations of 1000 individuals each: two source populations, an admixed population (75%/25% from each source population), and a fourth population. In Fig 3, we present PCs 1 and 2 coloured by population labels, k-means clusters, and UMAP-HDBSCAN($\hat{\epsilon }$) from two different replicates. In 50 replicates, HDBSCAN($\hat{\epsilon }$) identified four clusters corresponding largely to the population labels (e.g., Fig 3c) while in the other 50 the admixed population merges into a source population (e.g., Fig 3d). In contrast, k-means on the top 4 PCs with *K* = 4 returns consistent results (e.g., Fig 3b), though part of the admixed population is always clustered with a source population. We provide additional simulations in the supplement using a three-population out of Africa model as well as a 3×3 stepping-stone model with migration between neighbours. Illustrated in S4 Fig, the latter presents a scenario with both discrete and continuous population structure and highlights an important trade-off of the method: while it identifies the stepping-stone structure, the clusters (and UMAP visualizations) are highly-discretized representations of the data.

We use UMAP-assisted density-based clustering on data from three biobanks: the 1000 Genomes Project (1KGP), the UK biobank (UKB), and CARTaGENE (CaG). The 1KGP data consists of the genotypes of 3,450 individuals sampled from 26 populations from around the world; the populations were decided in advance and their sample sizes are similar, ranging from 104 to 183 individuals; this version of the 1KGP includes related individuals, which were not removed prior to analysis [23]. The UKB is a cohort of 488,377 individuals from the United Kingdom (UK) with genotypic, phenotypic, and sociodemographic data. UKB participants were recruited by inviting 9 million individuals registered with the National Health Service (NHS) who lived near a testing centre [24]. CaG is a cohort of residents of the Canadian province of Québec, with genotype data for 29,337 participants who were recruited using registration data from the Régie de l’assurance maladie du Québec (RAMQ), the provincial health authority, from four metropolitian areas in the province [25]. Unlike the 1KGP, CaG and the UKB do not have *a priori* populations defined, though they collected information about ethnicity, country of birth, and residential geographic distribution.

## Results

### Clustering captures population structure from sample design

**Fig 4:**
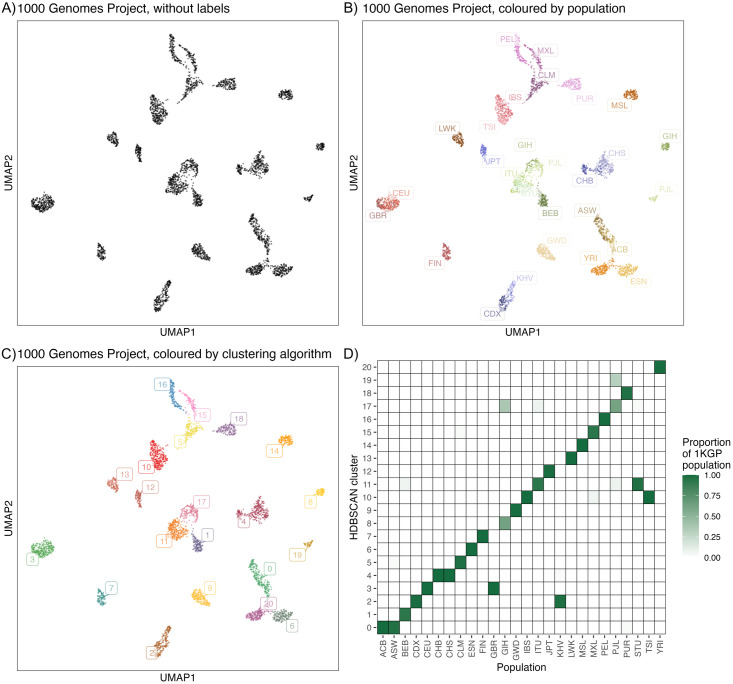
Clusters generated from 1KGP genotype data reflect its population sampling. **(A)** UMAP embedding of data without labels. **(B)** UMAP embedding of data, coloured by population label. **(C)** UMAP embedding of data, coloured by 21 clusters derived from HDBSCAN($\hat{\epsilon }$) applied to a 5D UMAP embedding. **(D)** Proportions of each 1KGP population contained within a given cluster. Population labels are provided in S1 Table. S5 Fig presents an alternative visualization using PCA instead of UMAP.

The 1KGP’s relatively balanced global sample design makes it useful for testing algorithms to identify population structure. We have previously shown that UMAP results in clear visual clusters from 1KGP data in two dimensions [4]. Fig 4 shows a UMAP representation of the 1KGP. Fig 4a shows the data without population labels (to mimic data with unknown populations), Fig 4b shows the data with corresponding population labels from the 1KGP, and Fig 4c shows the data with cluster labels generated by HDBSCAN($\hat{\epsilon }$) run on a 5*D* UMAP.

A major source of inhomogeneity in 1KGP data is its sampling scheme, which selected thousands of participants from 26 populations chosen based on a combination of geography, ethnicity, and ancestry criteria (e.g., Mexican ancestry in Los Angeles, USA). To provide a baseline for comparison, we calculated the Adjusted Rand Index (ARI) against 1KGP labels. These labels do not represent the truth about the populations, as we expect, e.g., population structure within the labelled populations. While a higher ARI with population labels does not mean a better clustering method, it does reflect an ability to capture a relevant source of heterogeneity (i.e., discrete sampling groups) within this particular dataset.

The clusters formed by UMAP and extracted by HDBSCAN($\hat{\epsilon }$) largely reflect this source of inhomogeneity, with some exceptions. Some pairs of populations are fully or almost-fully clustered together: GBR and CEU (British From England and Scotland; and Utah residents with Northern/Western European ancestry), CHB and CHS (Han Chinese in Beijing, China; and Han Chinese South, China), CDX and KHV (Chinese Dai in Xishuangbanna, China; and Kinh in Ho Chi Minh City, Vietnam), IBS and TSI (Iberian Populations in Spain; and Toscani in Italy), ITU and STU (Indian Telugu in the UK; and Sri Lankan Tamil in the UK), ACB and ASW (African Caribbean in Barbados; and African Ancestry in SW USA). While these groups differ in their sampling and history, supervised learning methods also merge most of these pairs (e.g., Fig 3A in [26]).

Five of these six merged 1KGP population pairs have the lowest pairwise-*F*_*ST*_ in the data (see S4 Table). Specifically, the CHB-CHS, CEU-GBR, and IBS-TSI pairs all fall fully into one cluster, while ITU-STU and CDX-KHV fall largely into one cluster with a small number of individuals clustering with others from the continental population. With ACB-ASW, all ACB individuals are part of Cluster 0 while four individuals are placed into clusters comprised largely of CLM, PEL, or MXL individuals—these latter individuals likely have more ancestry from the Americas, increasing the ACB-ASW *F*_*ST*_. We also note that the CDX and KHV (Cluster 2 in Fig 4b) populations are present at opposite ends of one continuous cloud of points. In other words, two groups belonging to one cluster does not mean that the groups are statistically indistinguishable. Rather, it means that HDBSCAN($\hat{\epsilon }$) could find a path in genetic space linking individuals sampled in one group to individuals sampled in the other, with each link based on local point density. Through a chain of such links, two individuals can be members of the same cluster even though they are not closely related.

Two groups GIH (Gujarati Indians in Houston, TX) and PJL (Punjabi in Lahore, Pakistan) are split into two clusters. These splits were observed previously in UMAP-based visualizations [4,27], and the GIH split was found to be strongly correlated with demographic identifiers (such as patronym) within the 23andme customer data, suggesting that it reflects some underlying demographic factor.

Fig 4d nevertheless shows strong agreement between population label and cluster label, (see also S2 and S3 Tables), with an ARI is 0.769. These results are comparable to a supervised neural network approach to predict sampled population label (e.g., Fig 3 in [26]), though our approach is unsupervised and runs much more quickly: depending on implementation, deriving the PCs can take 5–20 minutes, with the subsequent UMAP step requiring approximately 10 seconds and HDBSCAN($\hat{\epsilon })$ less than one second.

Though there have been methods developed to generate discrete population clusters from genetic data (e.g., [28]), most do not scale to hundreds of thousands of genomes. To provide a scalable basis for comparison, we considered an approach where k-means was performed on individual-level admixture proportions assuming *K* populations and then applied k-means clustering with *k* = *K* as the parameter. Using ADMIXTURE with *K* = 21 populations (to match the 21 clusters generated by HDBSCAN($\hat{\epsilon }$)), k-means clustering resulted in an ARI of 0.611, while *K* = 26 populations (to match the 26 1KGP populations) resulted in an ARI of 0.669 (see S7 and S8 Figs for visualizations, and methods for details). In another approach, we applied k-means clustering directly to the leading PCs; the best performing case was *K* = 25 on the top 20 PCs resulting in an ARI of 0.696 (S9 Fig).

**Fig 5:**
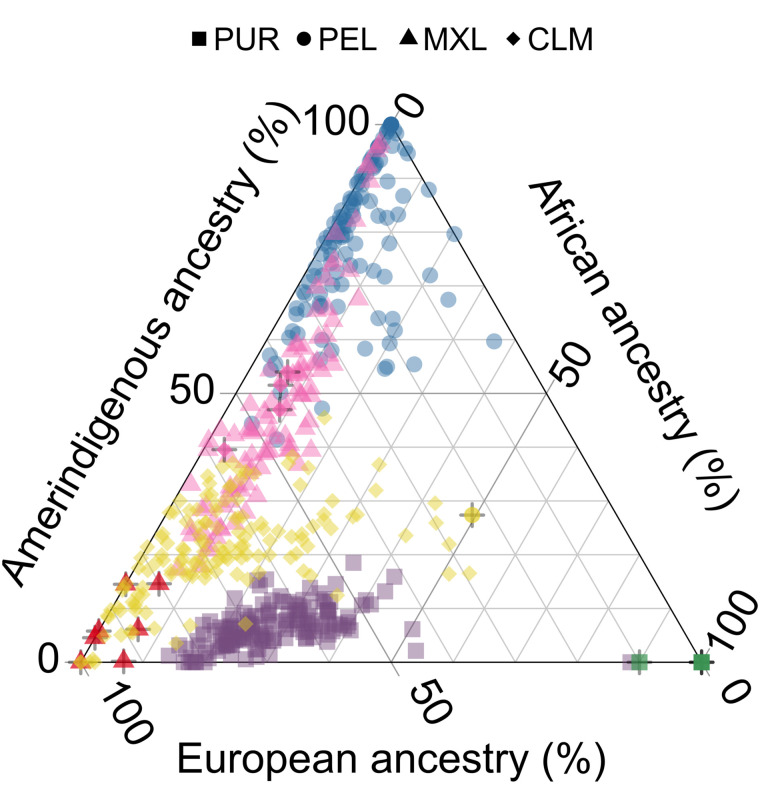
HDBSCAN clusters capture structure in populations with overlapping admixture proportions in the 1KGP. A ternary plot of the PUR, PEL, MXL, and CLM populations from the 1KGP with axes corresponding to global ancestry proportions estimated using ADMIXTURE (*K* = 3). Shapes indicate 1KGP label, colours indicate cluster label and match Fig 4c; bolded points with a + symbol indicate individuals who are not members of the modal cluster of their 1KGP population (full results given in S2 and S3 Tables).

One benefit of the unsupervised approach is that we do not require *a priori* assumptions about the origins of structure, making it possible to capture meaningful clusters despite considerable within-cluster heterogeneity, including in admixed populations. The admixed American population clusters largely match their 1KGP labels (CLM, MXL, PEL, PUR; ARI = 0.952), despite their heavily overlapping distributions in admixture proportions (illustrated in Fig 5). The ARI value is higher than the k-means-admixture approach, though k-means with *K* = 21 populations was close at ARI = 0.934 (see S8b Fig).

Changing parameters will influence cluster formation. Here we parametrized UMAP to preserve *n* = 50 neighbours, both for clustering and for visualization. In S6 Fig, we kept the same parameters for clustering, but used *n* = 15 neighbours for visualization. This resulted in some populations being split into different visual clusters. While some clusters will tend to persist across many parametrizations of UMAP and HDBSCAN($\hat{\epsilon }$), others based on more subtle patterns or in populations with more continuous variation will be less stable—although discrete groupings can help us understand data, the delineations are always, to a degree, arbitrary.

### Correlates between populations and sociodemographic, phenotypic, and environmental variables

The UK biobank (UKB) contains 488,377 genotypes from volunteers with an array of demographic, phenotypic, and biomedical data, with individuals’ ages at recruitment ranging from 40 to 69. The demographic data collected for the UKB include Country of Birth (COB) and Ethnic Background (EB), which is selected from a nested set of pre-determined options (see S6 Table). Participants first select their “ethnic group” from a list (e.g., “White”; “Black or Black British”), which determines the list of possible “ethnic background” values (e.g., “British”; “Caribbean”). The most common countries of birth in the data set are England, Scotland, Wales, and the Republic of Ireland, comprising 77.8%, 8.0%, 4.4%, and 1.0%, respectively. For EB, 88.3% of participants selected “White British”, with an additional 5.8% selecting “White Irish” or “Any other white background”. Here we primarily focus on the 28,814 individuals with other backgrounds.

Many studies of the UKB discard data from non-European individuals, sometimes citing concerns related to confounding from population structure [14]. The population structure has been deeply explored, though typically focused on British or European individuals [29–31]. Because its sub-populations are numerous, geographically/ancestrally diverse, and of widely varying sizes, clustering the UKB data is challenging, requiring overly broad categorization (e.g., a small number of continental populations [12,17]) and/or significant computational resources. The original implementation of HDBSCAN, without the $\hat{\epsilon }$ parameter, discards much of the UKB data as noise and splits populations into hundreds of microclusters that are not interpretable (S10 Fig).

**Fig 6:**
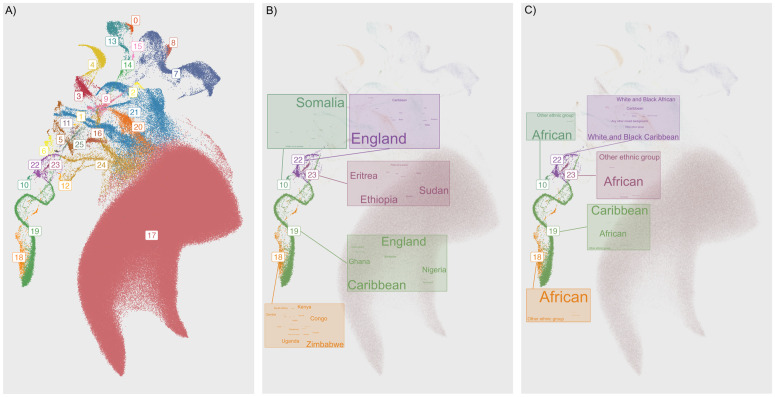
An example of clusters of population structure in the UKB. The clusters reflect a mixture of demographic history within the UK, the geographic origins of recent immigrants, the colonial history of the British Empire, and ongoing admixture. **(a)** Left: A 2D UMAP of UKB genotypes coloured by HDBSCAN($\hat{\epsilon }$). This parametrization generated 26 clusters. S21 Fig presents an alternative visualization using PCA. **(b)** Middle: Five clusters are highlighted with word clouds for the most common countries of birth within the cluster. **(c)** Right: The same five clusters are highlighted with word clouds for the most common EB within the cluster. Admixture proportions for clusters are presented in S12 Fig. Detailed breakdowns of EB and country of birth are presented in S7 and S8 Tables. An alternative clustering is presented in S22 Fig.

Fig 6a shows 26 clusters generated by HDBSCAN($\hat{\epsilon }$), placing 99.99% of individuals in clusters. We generated word clouds for COB and EB, shown in Figs 6b and 6c, which allow us to illustrate sources of structure without having to impose a label to groups which may be heterogeneous. Individuals in Cluster 10, for example, are mostly born in Somalia (84%), while those in Cluster 23 are mostly born in East Africa (Ethiopia, Sudan, Eritrea; 33%, 29%, 25%, respectively). Those in Cluster 18 are mostly born in African countries at or south of the equator, and 77% chose “African” as their EB, while 19% chose “Other ethnic group”. S11 Fig presents word clouds for another subset of data. Individuals in Cluster 0 are mostly born in Japan and South Korea (84% and 9%, respectively), and those in Cluster 15 are mostly born in Nepal (80%). In contrast, individuals in Cluster 13 are born in a variety of East/Southeast Asian jurisdictions; the most common EB was “Chinese” (70%), followed by “Other ethnic group” (16%) and “Any other Asian background” (11%). S7 and S8 Tables provide breakdowns for clusters.

Clusters 14 and 22 both capture structure resulting from recent admixture following immigration and colonial history, with 49% and 66% of their respective populations being born in England (see also S12 Fig). No single EB represents a majority in either cluster; the most common EB in Cluster 14 is “Any other mixed background” (29%), while for Cluster 22 it is “Mixed, White and Black Caribbean” (39%).

In clusters where the majority of individuals were born in Africa, large proportions chose “Other ethnic group” as their EB (24%, 19%, and 37% in Clusters 10, 18, and 2, respectively). Filtering data based on EB would reduce both genetic and ethnic diversity in a sample, and this reduction would be unequal between groups. Cluster 18 captures mostly individuals born in African countries at or south of the equator, while Cluster 19 consists of individuals born in the Caribbean (31%), England (28%), as well as Nigeria (14%) and Ghana (12%). These regions are historically linked to the UK; between the years 1641 and 1808, an estimated 325,311 Africans from the Bight of Benin, between the coasts of modern-day Ghana and Nigeria, were enslaved by the British and sent to the British Caribbean [32,33].

Despite the complexity of the UKB, topological clustering identifies population structure that is interpretable from historical or demographic perspectives and includes all or almost all individuals. Such structure is difficult to infer from a single label such as geography or ethnicity; once it is characterized, it can clarify the genetic structure of the cohort.

### Phenotype smoothing and modelling

Epidemiological research often focuses on observed differences between groups—for example, finding the mean of a phenotype or sociodemographic measure and comparing between populations. Clustering is one method to define groups based on shared genetic ancestry and compare means across groups. However, clustering data featuring continuous variation patterns can be sensitive to input parameters, and the sizes of clusters can vary across parametrizations, making it challenging to identify the “right” choice of parameters to test for heterogeneity.

One approach to visualize heterogeneity without relying on or overemphasizing a specific clustering is to average phenotypic mean values over multiple parametrizations. Mathematically, if *m*(*i*) represents the phenotype in individual *i* and *s*_*p*_(*i*) represents the set of individuals in the same cluster as *i* in parameterization p∈P, the cluster mean for *i* and parameterization *p* is $\mu_{p,i}=\frac{1}{\left|s_{p}(i)\right|}\sum_{j\in s_{p}(i)}m(j),$ and the smoothed value across parameterizations is


$$\mu_{i}:=\frac{\sum_{p\in P}\mu_{p,i}}{|P|}.$$ (1)

This is a somewhat ad-hoc smoothing approach, but it has clear benefits over standard approaches such as the conceptually related *k*-nearest-neighbour smoothing: the size and shape of averaging neighbourhoods are adapted to the topological structure of the data, enabling efficient smoothing across large clusters without smudging small ones. To illustrate this, we used Equation 1 on 288 clusterings of UKB data, which were the result of a grid search of UMAP and HDBSCAN($\hat{\epsilon }$) parameters (outlined in S1 Text).

**Fig 7:**
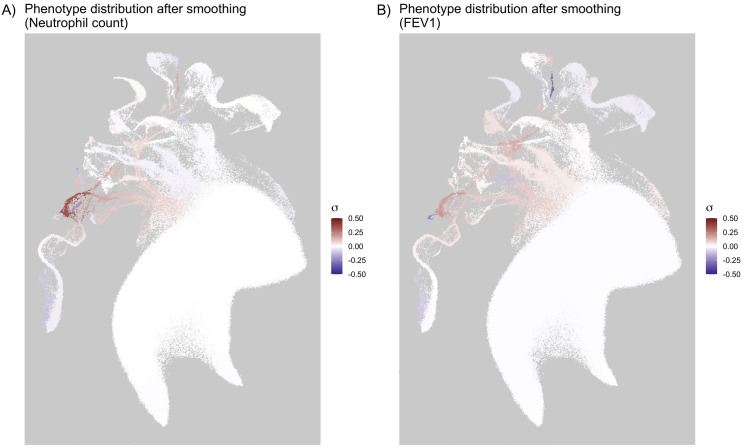
Smoothed phenotypic measures across multiple parametrizations of clustering. A 2D UMAP coloured by phenotype residuals after having regressed the top 40 PCs. Results were averaged by clusters, and we show averaged averages over 288 parametrizations of the clustering pipeline. The colour scale runs from −0.5σ to 0.5σ, for the standard deviation *σ* of each phenotype after regressing the linear effects of the top 40 PCs. We observe that the distributions of phenotypes among some groups are not centred about 0 even after PC adjustment. **(a)** Left: FEV1. **(b)** Right: Neutrophil count.

We visualize these smoothed values in Fig 7 for two phenotypes: FEV1 and neutrophil count. Despite having regressed out the effects of the top 40 PCs, there remains structure in the distribution of the residuals, visible at the scale of 0.5σ, where *σ* is the standard deviation of the phenotype across the UKB. For example, the average residual value is noticeably higher in individuals who fell in Cluster 22 as defined in Fig 6a. This cluster is composed mostly of individuals with admixed African/European backgrounds, and although they are intermediate in PCA space to African and European ancestry populations (S15 Fig), their phenotype distributions are not intermediate to clusters of primarily European- and African-ancestry individuals (S16 Fig, S17 Fig).

**Fig 8:**
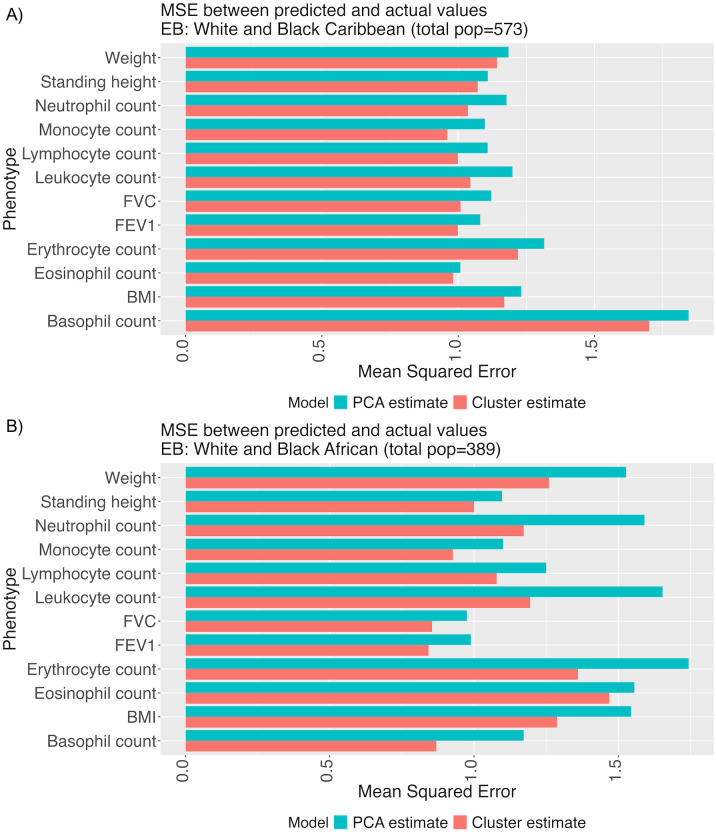
Cluster-based estimation can improve phenotype models. To test the explanatory value of smoothed cluster estimates generated from Equation 1, we carried out a five-fold cross-validation on the UKB data, compared phenotype prediction using the top 40 PCs versus estimates generated from the residual structure presented in Fig 7, and calculated the average MSE across folds.

To measure the explanatory value of these smoothed cluster estimates versus PC coordinates for these admixed individuals, we compared simple linear models for phenotype prediction using the top 40 PC coordinates for each individual versus using the smoothed estimates made from residuals after removing the effects of the top 40 PCs. We compared the models for populations that selected “Mixed” as their EB in the UKB questionnaire and found that for individuals who selected “White and Black Caribbean” (*n* = 573) or “White and Black African” (*n* = 389), the smoothed cluster estimates outperformed the PCA model, with an improved mean squared error across several phenotypes (see Fig 8; full table of MSE values in S11 and S12 Tables).

### Evaluating transferability of polygenic scores

Most investigations of PGS transferability are done at a population-level using large-scale geographical groups (e.g., “African”, “European”, “Asian”). However, these broad populations themselves exhibit population structure [34]. To illustrate the value for finer population groupings, we use our 26 cluster labels from Fig 6a, and compared the transferability of PGS across them.

**Fig 9:**
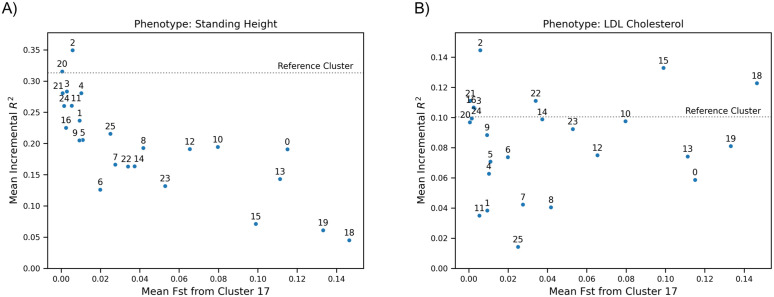
PGS accuracy by *F*_*ST*_ for standing height and LDL. A plot of the mean incremental *R*^2^ of a PGS against the difference in *F*_*ST*_ from the White British in the UKB. We use clusters extracted using HDBSCAN($\hat{\epsilon }$). **(a)** Top: A PGS of height shows a strong decay between *R*^2^ and *F*_*ST*_, as expected. **(b)** Bottom: A PGS of LDL-cholesterol has an unclear relationship between *R*^2^ and *F*_*ST*_. Cluster 18 has the largest *F*_*ST*_ but one of the highest *R*^2^ values; the cluster also has the highest frequency of the *rs*7412 and *rs*4420638 alleles.

Using UKB data, we estimated effect sizes of SNPs using VIPRS [35]. As a training population, we used individuals who selected “White British” as their EB to mimic the well-documented overrepresentation of European-ancestry individuals in GWAS. We estimated phenotypes for individuals and calculated the values of the fixation index (*F*_*ST*_) between the clusters (noting that *F*_*ST*_ between clusters defined using genotype data will be artificially inflated overall by the clustering process, see Discussion). In Fig 9, we plot the PGS accuracy for two phenotypes—standing height and low-density lipoprotein cholesterol (LDL)—against the *F*_*ST*_ for each cluster relative to Cluster 17, a cluster with over 400,000 individuals and with significant overlap with the training population (>95% selected “White British” as their EB). We observe for height (Fig 9a) that as the *F*_*ST*_ between populations grows, the predictive value of the PGS decreases; such a decrease is expected, due to factors like population-specific causal variants, gene-by-environment interaction, differences in allele frequencies, and linkage disequilibrium between assayed SNPs and causal variants [36].

However, we see no such relationship for LDL (Fig 9b). Cluster 18, composed mostly of individuals born in African countries at or south of the equator and of whom 77% selected the EB “Black African”, has one of the best PGS predictions despite its large *F*_*ST*_ from the training population. This may be because there are a few variants with large effect sizes; in contrast to height, LDL has been noted for its relatively low polygenicity [7]. Genome-wide *F*_*ST*_ represents average relative differentiation over a large number of variants. Differentiation at a single locus can differ appreciably from the genome-wide average. Under neutral evolution, the more variants control a trait, the more we expect that their average differentiation will reflect the genome-wide average.

To test if the frequencies of certain alleles impacted the PGS estimates, we modelled the *R*^2^ from the VIPRS estimates for each cluster against minor allele frequencies (MAF) of the top 100 SNPs and found the two strongest results were for *rs*4420638 and *rs*7412 (S9 and S10 Tables; S18 and S19 Figs, respectively). Both have their highest frequencies in Cluster 18 and both markers are in the apolipoprotein E (APOE) gene cluster; *rs*7412 had the largest overall effect size ($\hat{\beta }=-0.1812$), while *rs*4420638 had the second largest effect size in the opposite direction ($\hat{\beta }=0.02813$). The *rs*7412 allele has been linked to LDL [37] and was found to explain significant variation in LDL in African Americans [38]. The *rs*4420638 allele was associated with LDL even in the presence of the *rs*7412 allele in a study of Sardinian, Norwegian, and Finnish individuals [39]; it was also found to affect LDL in studies of children in Germany [40] and China [41].

The relationship between PGS accuracy and fine-scale population structure is complex and will vary by phenotype. It is not immediately obvious whether a PGS will transfer when there is a large degree of differentiation between the estimand and training populations. An approach like UMAP-HDBSCAN($\hat{\epsilon }$) can provide a detailed picture of the performance of a PGS in various genetic subgroups.

### Quality control for complex multi-ethnic cohorts

Population structure can be intricate. Demographic forces shaping it can be poorly understood, at least by geneticists analyzing a biobank. Individuals with uncommon combinations of ancestral, geographic, and ethnic descriptors are present in all biobanks. These combinations can be real and represent the completely different nature of genetic ancestry and ethnicity; they may also represent clerical errors [42]. Distinguishing the two is especially relevant when biobanks are used as sample frames for deeper sequencing or for follow-up studies, and when variables like country of birth and ethnicity are used as selection criteria. Using HDBSCAN$\left(\hat{\epsilon }\right)$ to explore the relationship between clusters membership and auxiliary variables can detect data collection errors before sample selection is carried out, preventing serious methodology problems or unnecessary exclusion of individuals.

**Fig 10:**
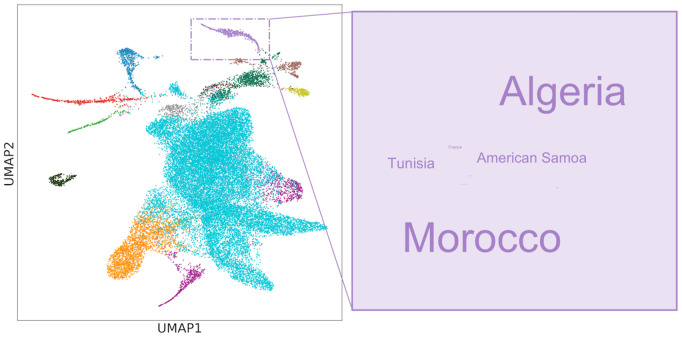
Clustering can identify data collection errors. A 2D UMAP of CARTaGENE data coloured by clusters extracted using HDBSCAN($\hat{\epsilon }$). The highlighted cluster was found to have most of its individuals born in North Africa. A word cloud shows that a significant minority of individuals were born in American Samoa, which was found to be a coding error.

CARTaGENE is a biobank of residents from Quebec, Canada, that has recently genotyped 29,337 individuals [43]. We were interested in identifying populations of North African descent for further study. In Fig 10, we identified a cluster of 446 people born largely in North Africa with 51 individuals (11.4%) recorded as being born in American Samoa, an American island territory in the South Pacific Ocean with fewer than 50,000 inhabitants. After researching possible historical explanations (e.g., migration between American Samoa and North Africa), we traced the result to a coding error from different country codes used over the course of data collection; the actual birth country was corrected to Algeria. The same coding error was found in other clusters, affecting 266 individuals born in 43 countries. While this error could have been discovered through other means, including detailed analysis of a population or other clustering techniques, the relative speed and flexibility of HDBSCAN($\hat{\epsilon }$) allowed for quick identification of an issue affecting less than 1% of the cohort. Efficient data exploration, aided by visualization and clustering, remains one of our best tools to combat the dual evils of bookkeeping errors and batch effects.

## Discussion

We present UMAP-HDBSCAN($\hat{\epsilon }$), a new approach to describe population structure that approximates the topology of high-dimensional genetic data and detects dense clusters in a low-dimensional space. Among its distinguishing characteristics:

1. All or almost all individuals are assigned to a cluster that correlates with demographic parameters.
2. It can be applied to biobanks with diverse compositions.
3. It does not depend on external labels, reference populations, or pre-defined boundaries. Researchers can generate clusters using genetic data and characterize them *post hoc* using either reference panels or auxiliary information, such as responses to questionnaire data.
4. It is highly tractable. The UKB provides pre-computed PCs, and running UMAP on the pre-computed top 40 PCs using a single core takes approximately 20 minutes; running HDBSCAN($\hat{\epsilon }$) on a UMAP embedding takes seconds. A grid search of parameters can be quickly executed on a computing array.

To our knowledge, no other clustering method has all of these characteristics; most are not able to meet the first criterion as they either fail to assign many individuals to a cluster or generate clusters that are uninformative of demographic history (e.g., through creating numerous micro-clusters or arbitrarily partitioning data).

The most commonly used approaches for fine-scale genetic community identification are based on measures of recent relatedness such as identity-by-descent (IBD; see, e.g., [8,9,44,45]). An IBD-based approach in ATLAS, for example, identified associations between genetic clusters and genetic, clinical, and environmental data [46]. The ability of IBD clustering to identify fine-scale structure can be due to two effects. First, it focuses on recent relatedness between individuals, which may be helpful in identifying recent demographic effects. Second, because it focuses on pairwise similarity, it encourages the use of clustering methods that focus on genetic neighbourhoods, i.e., on more topological approaches.

Despite the existence of such methods, researchers commonly rely on hand-delineation of dimensionally-reduced data (e.g., [2,12]). This is because IBD clustering is analytically demanding, and because IBD clusters focus on recent relatedness and may not reflect overall genetic similarity observed in PCA or UMAP plots. The topological approach presented here is meant to capture overall genetic similarity. Since it bypasses the need to perform phasing and IBD calling, it requires fewer analytical tools and computational resources. It can model populations of widely varying sizes and requires neither reference panels nor *a priori* definitions of populations, but can use auxiliary data such as geographic coordinates, jurisdiction, country of birth, population label, ethnicity, etc., to characterize the clusters *a posteriori* and learn about their history or origins. One limitation, however, is that at the moment there is no interpretation of the clusters generated in terms of population genetic theory. We also caution that statistical inference about clusters using the same data used to generate clusters (e.g., making inferences about *F*_*ST*_) will fail to control Type 1 error; this problem and its potential solutions are further detailed in [47].

A recent publication suggested moving entirely away from stratification based on genetic clusters [7]. Instead, they argued in favour of individual-level measures. They cite three issues with clusters: (i) clustering algorithms fail to capture populations without reference panels, such as those that are relatively small or recently admixed; (ii) clusters ignore inter-individual variation; and (iii) clustering results change based on algorithms and reference panels.

We believe that these criticisms are valid for the type of archetype-based stratification considered in [7]: if an individual fell within a certain PCA distance of one of nine pre-defined population centroids, they were considered a member of a cluster; otherwise, their ancestry was considered unknown. With this centroid-based method, 91% of participants in the UKB were placed into clusters in [7]. In contrast, across 604 runs of our topological approach with varying parameters, the median percentage of individuals placed in a cluster was 99.99% (S20 Fig), with the three runs with the largest number of unclustered individuals of UMAP-HDBSCAN($\hat{\epsilon }$) respectively assigning 99.11%, 99.69%, and 99.86% of individuals in the UKB to a cluster. Additionally, as the topological approach does not seek to minimize distance from a specific point (or “type”), a cluster does not imply limited inter-individual genetic variation among its constituent members. The clusters generated reflect groups that have shared genetic and geographic histories, including for relatively small and recently admixed groups which were often excluded based on prior approaches [7,15]. We achieved similar results with CaG and 1KGP data, suggesting that our approach is robust to details of biobank composition.

### Applications

Understanding the population structure of a biobank is a necessary precursor to many analyses. In the 1KGP, inhomogeneity in the data is heavily influenced by the sampling scheme, as reflected in Fig 4—the populations were deliberately sampled from multiple locations around the world with similar sample sizes. The sources of population structure of the UKB, on the other hand, reflect a complex history of migrations at different geographic and time scales, including isolation by distance within the UK and recent immigration and admixture.

The structure of a typical biobank is more similar to the UKB than the 1KGP, as the recruitment methodology is often based on residence within a jurisdiction. Examples include municipal (ATLAS in Los Angeles [46], Bio*Me* in New York City [48]), regional (CARTaGENE in Quebec [25]) and national (Million Veterans Project (MVP), [49], CANPATH [50]) biobanks. Leveraging these diverse cohorts can improve variant discovery [51,52].

Though population labels like ethnicity can be useful, individuals may identify as “Other” or “Unknown”, leading to incomplete data. In the MVP, missing data were imputed using a support vector machine trained on race/ethnicity data to harmonize genetic data with labels for an ethnicity-specific GWAS [53]. A similar supervised approach with random forests was used by gnomAD [18]. Rather than assigning ethnicities to individuals, we constructed clusters from genetic data and investigated the distributions of auxiliary variables within clusters, including missing values. We found word clouds to be well-suited for describing clusters without imposing a reductive label.

The goal of genetic stratification is in no way to replace variables such as ethnicity in contexts where they are relevant. In fact, genetic stratification revealed interesting trends in how participants selected these variables. For example, in Cluster 17 of Fig 6a, 97.6% of individuals were born in Britain and Ireland and 99.5% chose an ethnic group label; in contrast, 18.9% of those in Cluster 18 (mostly born in African countries at or south of the equator) and 36.5% in Cluster 23 (mostly born in the Horn of Africa) chose “Other”, highlighting differential completeness of questionnaire data. UKB strata with “mixed” ethnic backgrounds as their mode featured multiple ethnic background labels, likely reflecting both the fact that (genetically) admixed individuals may have a diversity of ethnic backgrounds, and the fact that individuals with both mixed genetic and cultural heritage may have to choose among potentially inadequate labels (see, e.g., discussion in [15]). The presence or absence of a label in data collection can itself critically influence how people identify: between the 2011 National Household Survey and the 2016 Census in Canada, there was a 53.6% drop in people who identified as “Jewish” because the label was not provided as an example ethnicity in the 2016 questionnaire [54].

### Considerations about topological clustering

In this work, we have used UMAP for two different purposes: visualization, and clustering. Despite some criticisms of UMAP visualizations [55], it remains a popular tool [56,57]; our choice to use UMAP for visualization of clusters in this work is not central to our conclusions, and we have provided many alternative visualizations using PCA. By contrast, we have found that using UMAP in moderate dimensions (usually 3–5) was an important preprocessing step for improving HDBSCAN($\hat{\epsilon }$) performance. Increasing the UMAP dimensions from 2 allows for better preservation of the original neighbourhoods [55] which is expected to alleviate (though not eliminate) many of the drawbacks observed in UMAP in 2D.

Unlike archetype-based methods that invite essentialism [58,59], HDBSCAN($\hat{\epsilon }$) emphasizes relationships among samples by identifying groups that can be created by linking nearby individuals—it is possible to have a long chain containing many individuals who are each closely related to those near them within a cluster but not to those at the distant end. Admixed populations can form a single cluster even though individuals within clusters can differ as much as individuals from the different ancestral “source” populations. HDBSCAN($\hat{\epsilon }$) identifies groups of individuals with a continuous distribution in genetic space and degree of separation from other clusters in a given dataset. Separation in this case can originate from natural causes (such as reproductive barriers) or sampling effects (such as discrete sampling in a continuous population). This can be both a strength and a limitation of the approach depending on purpose. However, given the weaponization of population genetics research in the past [60], it is worth emphasizing limitations common to all clustering approaches.

No single label is an individual’s “true” ancestry, race, or ethnicity, as these are complex, multifactorial population descriptors [15,61]. Thus clustering does not have a well-defined ground truth [62], and clusters are most useful as “helpful constructs that support clarification” [63]. With real genetic data, there is no “correct” number of populations [64] and discrete groupings provide a flattened view of a high-dimensional landscape [15,65]. The clusters generated are sensitive to the input data—since the demographic composition of a biobank will impact the clustering,—and they are also affected by the parameters at the filtering, dimensionality reduction, and clustering steps. This is a reflection of the data, as genetic data are not composed of “natural types”. These clusters can be useful in understanding how genetics relates to health and the environment, but variation in phenotypes across genetic clusters does not imply a genetic cause, as differences in environment or systemic discrimination are also expected to produce such variation [66]. The identification of topological clusters does not imply an absolute inter-cluster distance (e.g., different clusters can have low *F*_*ST*_), nor within-cluster similarity (e.g., pairs of individuals within a cluster can be distant). The UK biobank clusters of majority African-born individuals, for example, each encompass considerable genetic substructure [67]. Different choices of metrics for clustering (i.e., genetic relatedness vs. IBD) can emphasize different types of structure. There are no true clusters.

Ultimately, however, many useful analyses require some definition of “populations”. For example, an allele frequency can only be calculated and reported within a specific set of individuals. Data exploration and quality control often require investigating relevant subsets of the data to decide whether they reflect technical artefacts or meaningful subgroups. To date there has not been a method of stratification that is tractable, easy to implement, able to recognize clusters of many sizes, and that captures all or almost all individuals with complex population histories. We believe our topological approach satisfies these important needs. Looking forward, we expect that topological approaches underlying UMAP and HDBSCAN($\hat{\epsilon }$) also present a promising avenue to move towards a more continuous description of genetic variation in complex cohorts.

### Method details

Our code is available at https://github.com/diazale/topstrat. We have provided command line tools to run Python implementations of UMAP and HDBSCAN($\hat{\epsilon }$). We used three datasets for this analysis: the 1000 Genomes project (1KGP), the UK biobank (UKB), and CARTaGENE (CaG). Simulation data are available on Zenodo at https://doi.org/10.5281/zenodo.17545804. Code used to generate simulation data is available at https://github.com/hmsnell/topstrat_popsims.

For the 1KGP we used 3,450 genotypes using Affy 6.0 genotyping [23]; this dataset contains related individuals. We generated the PCs using a Python script and have made the top PCs available in the repository to demonstrate the code. We used the genotype file

ALL.wgs.nhgri_coriell_affy_6.20140825.genotypes_has_ped.vcf.gz
and population labels
affy_samples.20141118.panel 20131219.populations.tsv,

available at http://ftp.1000genomes.ebi.ac.uk/vol1/ftp/release/20130502/supporting/hd_genotype_chip/. We generated admixture proportions using ADMIXTURE 1.3.0 [68] from 45,197 SNPs. Using 32GB of RAM and 32 cores, this took 10,554 seconds to run with *K* = 21 populations and 40,719 seconds to run with *K* = 26 populations.

For the UKB, we limited our analyses to the 488,377 individuals with genotype data. We used the UKB’s top 40 pre-computed PCs (Data-Field 22009), blood cell counts (Data-Fields 30000, 30010, 30120, 30130, 30140, 30150, 30160), lung function measures (Data-Fields 3062, 3063), age (Data-Field 21003), sex (Data-Field 31), standing height (Data-Field 50), weight (Data-Field 21002), BMI (Data-Field 21001), smoking status (Data-Field 20116), country of birth (Data-Fields 1647, 20115), and ethnic group/background (Data-Field 21000). Since the top PCs were pre-computed, we did not include them in our runtimes. Ethnic group/background is a hierarchical item in which participants are prompted to select from a pre-populated list of options for Ethnic Group (e.g., “White”) and, if available, a secondary option for Ethnic Background (e.g., “British”). Phenotypes used in smoothing analyses were normalized with respect to variables *sex*, *age*, and *age*^2^. Access to the UKB can be granted at https://www.ukbiobank.ac.uk/scientists-3/genetic-data/.

For CARTaGENE, we used 29,337 individuals with genotype data. We generated the PCs using PLINK [69–76] after filtering for linkage disequilibrium and HLA (chromosome 6, 25000000–33500000). The options used were:

- indep-pairwise 1000 50 0.1 (PLINK2)
- maf 0.05
- mind 0.1
- geno 0.1
- hwe 1e-6.

We used the Python implementations of UMAP [20] (0.3.6) and HDBSCAN (0.8.24), integrating the updates from Malzer and Baum [19].

To calculate PGS, we used VIPRS [35]. Phenotypes were residualized by age, sex, and PCs. We present the incremental R-squared generated by VIPRS, which is calculated as follows:

1. Fit two linear models (Full and null)
  - Full: *y* = *PGS* + *covariates*
  - Null: *y* = *covariates*
2. Compute the incremental *R*^2^ by computing the difference in the *R*^2^ of the two models: $R^{2}=R^{2}(Full)-R^{2}(Null)$

## Supporting information

S1 Text Supporting information on algorithm parameters and simulations. (PDF)

S1 FigHDBSCAN($\hat{\epsilon }$) on simulated out of Africa (OOA) data.We ran UMAP and HDBSCAN($\hat{\epsilon }$) on 100 replicates of simulated OOA data for three populations. In 94 replicates, the algorithm identified three clusters, retrieving the discrete structure. **(a)** The replicate with the highest ARI, coloured by population label (considered ground truth here, though different ground truths could be considered) **(b)** The replicate with the highest ARI, coloured by HDBSCAN($\hat{\epsilon }$) cluster. The cluster labels were robust across parameter values for minimum points and $\hat{\epsilon }$ and largely match population labels that were simulated, retrieving the discrete structure.(PNG)

S2 FigVarying parameters in HDBSCAN($\hat{\epsilon }$).We use the OOA simulation from S1c Fig. **(a)** Varying $\hat{\epsilon }$ while holding the minimum points fixed. Lower values result in more clusters forming as small clusters no longer merge, but result in more noise points. **(b)** Varying minimum points while holding $\hat{\epsilon }$ fixed. The high $\hat{\epsilon }$ minimizes noise points by merging clusters; small (minimum size 10) and large (minimum size 50) clusters are merged, while the medium cluster (minimum size 25) is stable and does not merge.(PNG)

S3 FigSimulations of admixed data.Simulated data of two source populations, an admixed population (with 25%/75% ancestry from the sources) and a fourth population. **(a)** UMAP of a replicate coloured by population label (considered ground truth here, though different ground truths could be considered). **(b)** The replicate from (a) coloured by HDBSCAN($\hat{\epsilon }$) returns four populations, reflecting discrete structure. **(c)** A different replicate, coloured by ground truth. **(d)** The replicate coloured by HDBSCAN($\hat{\epsilon }$); this time the algorithm merges two clusters. **(e)** PCA of the data, coloured by ground truth. **(f)** PCA, coloured by k-means (*K*=4). The box highlights how k-means groups part of the admixed population with a source population.(PNG)

S4 FigUMAP-HDBSCAN($\hat{\epsilon }$) and PCA identify the dual discrete-continuous nature of a stepping-stone model with continuous migration.Simulated data of a stepping-stone model with nine populations of 500 with continuous migration. **(a)** UMAP coloured by population label (considered ground truth here, though different ground truths could be considered) **(b)** UMAP coloured by HDBSCAN($\hat{\epsilon }$) clusters. **(c)** PCA coloured by ground truth. In this data, population structure manifests as both discrete (from the stepping-stones) and continuous (from migration). **(d)** The replicate with the lowest ARI when comparing ground truth to HDBSCAN($\hat{\epsilon }$)  coloured by ground truth. **(e)** The replicate from (d) coloured by HDBSCAN($\hat{\epsilon }$). Here the algorithm identifies some of the discrete structure, though it links many of the populations together from their migration. **(f)** The number of clusters identified across replicates.(PNG)

S5 FigTop 2 PCs of 1KGP data.**(a)** No colours, to simulate unknown populations. **(b)** Coloured by population label. **(c)** Coloured by clusters derived from HDBSCAN($\hat{\epsilon }$). These are the same as in Fig 4c.(PNG)

S6 FigAlternate UMAP visualization to illustrate how UMAP parameters can impact cluster formation.The UMAP used for visualization in Fig 4 was set to 50 neighbours. In this figure, we use 15 neighbours for the UMAP visualization, resulting in smaller visual clusters; the HDBSCAN($\hat{\epsilon }$) parameters are unchanged from the main text.(PNG)

S7 Fig Clustering 1KGP data using ADMIXTURE 1.3.0. (a) Generating 21 clusters using k-means clustering on admixture proportions (*K*=21 populations specified).
(PNG)

S8 Fig Adjusted Rand Indices (ARI) comparing hard clustering of global ancestry estimates. (PNG)

S9 Fig Adjusted Rand Indices (ARI) comparing k-means clustering of the top 20 PCs of the 1KGP data. (PNG)

S10 FigAn example of a clustering of the UKB data using HDBSCAN rather than HDBSCAN($\hat{\epsilon }$).The algorithm fails to cluster many of the sub-populations, categorizing 4,197 individuals as noise and generated almost 200 micro-clusters.(PNG)

S11 Fig Word clouds generated from four clusters in the UKB from Fig 6. (**a**) Left: Word clouds of the most common countries of birth within each cluster. Most individuals in the orange cluster (Cluster 0) were born in Japan, and most in the pink cluster (Cluster 15) were born in Nepal. (**b**) Right: Word clouds for the most common EB. The most common in the blue cluster (Cluster 13) was “Chinese”, while those in the green cluster (Cluster 14) select a variety, including “White British”, “Chinese”, “Mixed”, or “Other”. Detailed breakdowns are available in S7 and S8 Tables.
(PNG)

S12 Fig Admixture proportions for *K*=5 populations on each of the clusters in Fig 6. Cluster 17 (*n*>400,000) was excluded for computational reasons. Individuals not assigned to a cluster are labelled as −1.
(PNG)

S13 Fig FEV1 values averaged by a single run of clustering rather than smoothed over multiple runs of clustering. (JPEG)

S14 Fig Proportion of daily smokers, smoothed using Equation 1. (JPEG)

S15 Fig Cluster 22 from Fig 6a highlighted coloured in on a plot of PC1 and PC2. (PNG)

S16 Fig Distributions of FEV1 adjusted for age and sex stratified by cluster. Vertical dotted lines represent the mean of the distribution. Cluster labels and colours match those in Fig 6a. Cluster 17 is mostly European-born individuals; Cluster 18 is mostly individuals born in African countries at or south of the equator; Cluster 19 is mostly individuals born in England, the Caribbean, Ghana, and Nigeria; and Cluster 22 is mostly individuals born in England who chose the EB “White and Black Caribbean” or “White and Black African”. (a) Top: Distribution of FEV1 by cluster without adjusting for population structure. (b) Bottom: Distribution of FEV1 by cluster after having adjusted for the top 40 PCs.
(JPEG)

S17 Fig Distributions of neutrophil count adjusted for age and sex stratified by cluster. Vertical dotted lines represent the mean of the distribution. Cluster labels and colours match those in Fig 6a. Cluster 17 is mostly European-born individuals, Cluster 18 is mostly individuals born in African countries at or south of the equator, Cluster 19 is mostly individuals born in England, the Caribbean, as well as Ghana, and Nigeria in Western Africa, and Cluster 22 is mostly individuals born in England who chose the EB “White and Black Caribbean” or “White and Black African”. (a) Top: Distribution of neutrophil count by cluster without adjusting for population structure. (b) Bottom: Distribution of neutrophil count by cluster after having adjusted for the top 40 PCs.
(JPEG)

S18 Fig Regression line of the *R*^2^ of a PGS generated by VIPRS versus the minor allele frequency *rs*4420638, labelled by clusters from Fig 6a. The regression summary is presented in S9 Table.
(PNG)

S19 Fig Regression line of the *R*^2^ of a PGS generated by VIPRS versus the minor allele frequency *rs*7412, labelled by clusters from Fig 6a. The regression summary is presented in S10 Table.
(PNG)

S20 FigCounts of clustered individuals across multiple runs.For 604 runs of UMAP-HDBSCAN($\hat{\epsilon }$) on the UKB, we count the number of individuals not assigned to a cluster. (a) Top: Across all 604 runs. (b) Bottom: To improve the scale of the figure, we remove 3 outlier runs in which 684, 1,535, and 4,346 individuals were not assigned to a cluster.(PNG)

S21 FigTop 2 PCs of the UKB coloured by HDBSCAN($\hat{\epsilon }$).These are the same clusters used in Fig 6a.(PNG)

S22 Fig An alternative clustering of UKB data. Compared to Fig 6a, the largest cluster (Cluster 17 in that figure) has been split into three smaller clusters (Clusters 14, 24, 25 in this figure). Other clusters have been split or merged, while some remain the same between runs.
(PNG)

S1 Table Names and abbreviations of 1KGP populations. (XLSX)

S2 Table Cluster assignments for each 1KGP population, showing how many individuals from each population ended up in each cluster. (XLSX)

S3 Table Composition of each cluster broken down by 1KGP population. (XLSX)

S4 Table The lowest pairwise *F*_*ST*_ values for 1KGP populations. (XLSX)

S5 TableHDBSCAN($\hat{\epsilon }$) carried out on the top PCs of the UKB for varying values of the minimum number of points, $\hat{\epsilon }$, and the number of input PCs.(XLSX)

S6 Table Possible values for ethnic background in the UKB (Data-Field 21000). Participants are first asked “What is your ethnic group?” and then asked “What is your ethnic background?” For “Chinese”, there is no second question. Participants may also select “Prefer not to answer” for the second question; it is possible to have ethnic background recorded as ethnic group (e.g. just “White” or “Mixed”). Excluding “Do not know”, “Prefer not to answer”, and “Not available”, there were 20 unique values of ethnic background.
(XLSX)

S7 Table Frequency of country of birth by cluster for Fig 6a. Proportion refers to the proportion within the cluster. Categories with proportion below 0.05 are not listed.
(XLSX)

S8 Table Frequency of selected EB by cluster for Fig 6a. Proportions refer to the proportion within the cluster. Categories with proportions below 0.05 are not listed.
(PNG)

S9 Table Linear regression model of MAF and PRS *R*^2^. The model is between minor allele frequency (MAF) of *rs*4420638 within each cluster from Fig 6a and the *R*^2^ of a PGS for LDL generated by VIPRS using the clusters from Fig 6a. The plot of the regression is present in S18 Fig.
(XLSX)

S10 Table Linear regression model of MAF and PRS *R*^2^. Linear regression model between minor allele frequency (MAF) of *rs*7412 within each cluster from Fig 6a and the *R*^2^ of a PGS for LDL generated by VIPRS using the clusters from Fig 6a. The plot of the regression is present in S19 Fig.
(XLSX)

S11 Table Comparing two phenotype models split by EB. One model (PCA) uses the top 40 PCs to estimate phenotypes, while the other (CLS) uses a cluster-smoothed phenotype estimate from Equation 1 in addition to the top 40 PCs. Values in the table are the average mean squared error with the average number of testing samples in a five-fold cross validation.
(XLSX)

S12 Table Comparing two phenotype models split by EB. One model (PCA) uses the top 40 PCs to estimate phenotypes, while the other (CLS) uses a cluster-smoothed phenotype estimate from Equation 1 in addition to the top 40 PCs.Values in the table are the average mean squared error with the average number of testing samples in a five-fold cross validation.
(XLSX)
