# Supplementary material for: Topological stratification of continuous genetic variation in large biobanks
Source: PLoS Genet. 2026 Mar 16;22(3):e1012068. doi: 10.1371/journal.pgen.1012068 (PMC13008251; doi:10.1371/journal.pgen.1012068)
Supplement: S1 Text — (PDF) [file pgen.1012068.s001.pdf]

Supporting Information (SI)

Topological stratification of continuous genetic variation in large biobanks

Alex Diaz-Papkovich, Shadi Zabad, Hannah Snell, Chief Ben-Eghan, Luke Anderson-Trocmé, Georgette Femerling, Vikram Nathan, Jenisha Patel, Simon Gravel

For visualization, we reduce our data to 2D via UMAP and set a relatively high minimum distance (MD; usually between 0.3 and 0.5); this enables us to view fine-scale patterns of structure. We find satisfactory results with the number of neighbours (NN) varying from 15 to 50; higher values will require more computational resources, but they increase the connectivity between points in the data, as discussed in [1]. For clustering, we set a low value of minimum distance (equal to or close to 0) and reduce the number of dimensions to at least 3—in our analyses, we used 3, 4, and 5 dimensions. Both the low minimum distance and the higher dimensionality reduce the number of constraints on the points in the lower-dimensional space, making it easier to place the point without disrupting neighbourhoods from the original space.

The number of neighbours will vary depending on what is a reasonable expectation for the data. For the 1KGP data, which consists of geographically diverse samples of roughly similar size, 50 neighbours capture the structure well. For biobank data, it is common for structure to arise from a handful of individuals; we found 10 to 25 neighbours to work best. Lower neighbourhood values (e.g. $NN = 5$) will create smaller clusters, but can also highlight highly-localized structure within larger populations. $2D$ visualizations can give intuition as to the presence and sizes of clusters. If pre-processing the data with PCA, more PCs tend to reveal finer-scale structure (see e.g. the relationship with geographical coordinates in S17 and S18 Figs in [2]). For the 1KGP clusters in Fig 4 we used the top 16 PCs; for the UKB in Fig 6a and CaG in Fig 10 we used the top 25.

In parametrizing HDBSCAN($\hat{\epsilon}$), the parameter $\hat{\epsilon}$ defines a threshold at which clusters are merged or split. We find values of $\hat{\epsilon}$ ranging from 0.3 to 0.5 to be effective at ensuring all or almost all individuals are clustered while still identifying fine-scale structure. The minimum number of points (MP) should not be significantly higher than the number of neighbours used in the associated UMAP. If MP is high and NN is low, it can result in a large number of points being classified as noise since the UMAP data will tend to form small clusters; e.g. a UMAP parametrized with $NN = 10$ and HDBSCAN($\hat{\epsilon}$) with $MP = 100$ may return poor results.

Changing parameters may result in different clusters being generated. Given the low computational costs of UMAP and HDBSCAN($\hat{\epsilon}$), we recommend running a grid search for visualization and exploratory analysis. Clusters can then be characterized using auxiliary data, such as country of birth, geographical location, population label, self-identification, etc. We selected the clustering for the UKB for its suitability for comparing PGS results by F_{ST} from the training population. For CaG, we selected one of the clustering runs that generated a cluster of individuals with North African ancestry.

Pairwise F_{ST} for UKB clusters was calculated based on chromosome 1. We present the Hudson estimator calculated using PLINK2 [3, 4]. We calculated admixture proportions using ADMIXTURE 1.3.0 [5]. For computational reasons, for the UKB we

calculated admixture proportions on individuals not falling into Cluster 17 (the largest cluster, containing around $400,000$ individuals) in Fig 6a.

Visualizations and statistical analyses were done in R (3.5.3) [6]. We used ggplot2 [7] for graphics and ggwordcloud for word clouds,and stargazer [8] to generate tables.

For phenotype smoothing, we removed the effects of the top 40 PCs using linear regression, working with the residuals. For phenotype p and individuals $i = 1 \dots I$, we use the model:

$$y_{p,i} = \beta_{p,0} + \sum_{j=1}^{40} \beta_{p,j} PC_{j,i} + \epsilon_{p,i}, \epsilon_{p,i} \sim N(0, \sigma_p^2)$$

We visualize the data in Fig 7 with the values $e_{p,i} = y_{p,i} - (\hat{\beta_{p,0}} + \sum_{j=1}^{40} \hat{\beta_{p,j}} PC_{j,i})$

For UKB figures we varied the number of input PCs ($5 \dots 40$) into UMAP as well as the UMAP parameters (setting the number of neighbours to 5 or 10, the minimum distance to 0 or 0.01, and the dimensionality to 3 or 5) and set the HDBSCAN($\hat{\epsilon}$) parameters to 25 minimum points and $\hat{\epsilon}$ to 0.5. This resulted in $36 \times 2 \times 2 \times 2 = 288$ unique runs of parameters. To test the robustness of the clustering (shown in S20 Fig), we re-ran a subset of the parametrizations for a total of 604 runs. Starting from UMAP data, clustering the UKB took approximately 60 seconds on a single core.

Simulations

To give intuition to the method and compare to known population labels, we carried out a series of simulations using SLiM [9] based on the `stdpopsim` library [10]:

- A three-population out-of-Africa (OOA) model. Each population contained $1,000$ samples and simulated genotypes had an average of $11,200$ variants.
- An admixture model with four populations: two source populations, an admixed population with 25%/75% ancestry from the source populations, and a separate fourth population, with an average of $250,000$ variants.
- A nine-population 3×3 stepping-stone model with equal constant migration between neighbours. Each population contained 500 samples and simulated genotypes had an average of $17,000$ variants.

We generated 100 replicates for each model to see if HDBSCAN($\hat{\epsilon}$) could identify the broad discrete structure in the model, and compared population labels to cluster labels using adjusted Rand index (ARI). Unless otherwise stated, UMAPs in these analyses were performed on the raw genotype data.

In the OOA model, we fixed $\hat{\epsilon} = 0.5$ and the minimum points to 50; 94 replicates returned three clusters, correctly identifying the discrete population structure, with an average ARI of 0.938. In each of the 6 cases that did not return 3 clusters, two populations were merged into one. We present examples of both cases in S1 Fig. We highlight one replicate in S1d and S1e Figs to demonstrate how changing the minimum number of points for HDBSCAN($\hat{\epsilon}$) changed the number of populations.

In S2 Fig we show how varying parameters in the replicate from S1c Fig changes the clustering. Fixing a low minimum number of points (e.g. 10) while lowering $\hat{\epsilon}$ results in many small clusters being formed since the algorithm requires higher local point densities (and as $\hat{\epsilon} \to 0$ clusters will no longer merge). Consequently, it increases the number of noise points (which lay between clusters). Fixing $\hat{\epsilon}$ while increasing the minimum number of points in a cluster will vary the number of clusters. In these simulations, a small value for minimum points will require a higher $\hat{\epsilon}$ to avoid creating excessive noise points. In the OOA replicate just discussed, the many small clusters at

10 minimum points generate hundreds of noise points as $\hat{\epsilon} \to 0$; however, increasing the minimum points to 25 closely matches the population label, and this was robust to values of $\hat{\epsilon}$. For a more thorough discussion of these parameters, we refer the reader to [11].

In the admixture model, we ran HDBSCAN($\hat{\epsilon}$) on UMAP data, again fixing $\hat{\epsilon} = 0.5$ and setting minimum points to 50. The average ARI was 0.846. In half of the replicates we identified four clusters, while the remaining half connected and merged the admixed population with a source population, which we demonstrate in S3 Fig. This may be a consequence of the underlying UMAP parameter for neighbourhood size (25) being large enough to create connections between populations. Here too the results were robust to varying $\hat{\epsilon}$ values. We also provide PCA with k-means in S3f Fig. K-means results are consistent across replicates, but always split off some of the admixed population and group it with a source population. Finally, pre-processing data with PCA before running UMAP can change the clustering behaviour. For example, running UMAP on the top 4 PCs resulted in four clusters being extracted in all 100 replicates, with an average ARI of 0.99, in this simplistic example. Overall, we find that HDBSCAN($\hat{\epsilon}$) clustering can be sensitive to both choice of parameters and sampling noise, which is a clear downside relative to the robustness of k-means. Despite this sensitivity, HDBSCAN($\hat{\epsilon}$) produces cluster boundaries that align with the real cluster boundaries, by contrast with k-means which consistently produces an artificial boundary.

In the stepping-stone simulation, we ran HDBSCAN($\hat{\epsilon}$) on UMAP, fixing $\hat{\epsilon} = 0.5$ and setting minimum points to 100 resulting in an average ARI of 0.683. The average number of clusters found was 8.34. We highlight in S4a–S4c Figs how UMAP-HDBSCAN($\hat{\epsilon}$) and PCA can identify the dual discrete-continuous nature of the stepping stone model, where topological clustering identifies the island structure.

Simulation data are available on Zenodo at `https://doi.org/10.5281/zenodo.17545804`. Code used to generate simulation data is available at `https://github.com/hmsnell/topstrat_popsims`. We have provided a companion notebook to explore the simulations at `https://github.com/diazale/topstrat_simulation_companion`.

References

1. A. Diaz-Papkovich, L. Anderson-Trocmé, S. Gravel, A review of UMAP in population genetics, *Journal of Human Genetics* **66**, 85 (2021).

2. A. Diaz-Papkovich, L. Anderson-Trocmé, C. Ben-Eghan, S. Gravel, UMAP reveals cryptic population structure and phenotype heterogeneity in large genomic cohorts, *PLoS Genetics* **15** (2019).

3. C. C. Chang, *et al.*, Second-generation PLINK: rising to the challenge of larger and richer datasets, *GigaScience* **4**, s13742 (2015).

4. C. Chang, S. Purcell, PLINK2, `https://www.cog-genomics.org/plink/2.0/`.

5. D. H. Alexander, J. Novembre, K. Lange, Fast model-based estimation of ancestry in unrelated individuals, *Genome Research* **19**, 1655 (2009).

6. R Core Team, *R: A Language and Environment for Statistical Computing*, R Foundation for Statistical Computing, Vienna, Austria (2018).

7. H. Wickham, *ggplot2: Elegant Graphics for Data Analysis* (Springer-Verlag New York, 2016).

8. M. Hlavac, stargazer: Well-Formatted regression and summary statistics tables (2018).

9. B. C. Haller, P. W. Messer, SLiM 4: Multispecies Eco-Evolutionary Modeling, *The American Naturalist* **201**, E127 (2023).

10. J. R. Adrion, *et al.*, A community-maintained standard library of population genetic models, *eLife* **9**, e54967 (2020).

11. C. Malzer, M. Baum, A Hybrid Approach To Hierarchical Density-based Cluster Selection, *2020 IEEE International Conference on Multisensor Fusion and Integration for Intelligent Systems (MFI)* pp. 223–228 (2020).
