# Supplementary figures and images for: Topological stratification of continuous genetic variation in large biobanks
Source: PLoS Genet. 2026 Mar 16;22(3):e1012068. doi: 10.1371/journal.pgen.1012068 (PMC13008251; doi:10.1371/journal.pgen.1012068)

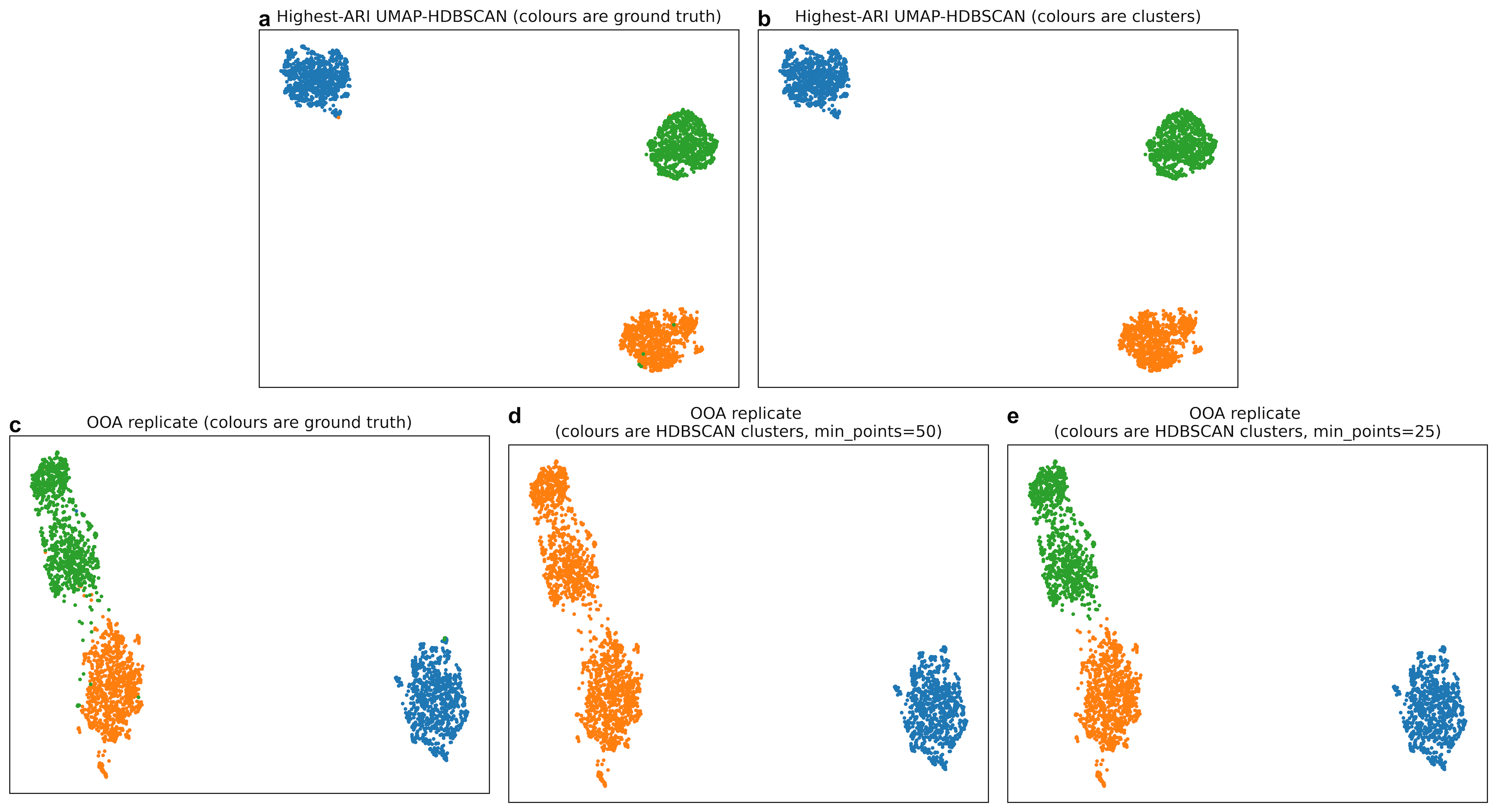

Supplement: S1 Fig — We ran UMAP and HDBSCAN(ϵ^) on 100 replicates of simulated OOA data for three populations. In 94 replicates, the algorithm identified three clusters, retrieving the discrete structure. (a) The replicate with the highest ARI, coloured by population label (considered ground truth here, though different ground truths could be considered) (b) The replicate with the highest ARI, coloured by HDBSCAN(ϵ^) cluster. The cluster labels were robust across parameter values for minimum points and ϵ^ and largely match population labels that were simulated, retrieving the discrete structure. (PNG) [file pgen.1012068.s002.png]

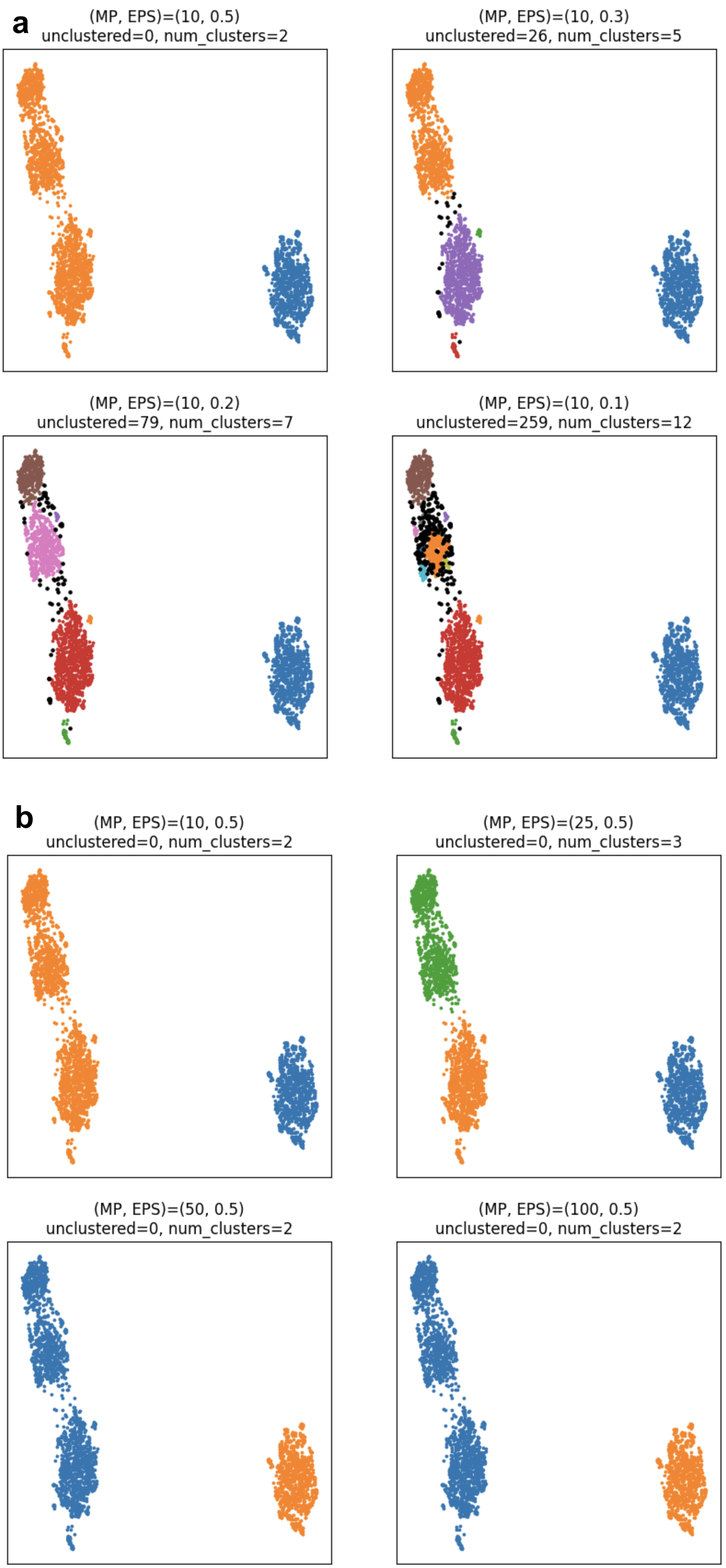

Supplement: S2 Fig — We use the OOA simulation from S1c Fig. (a) Varying ϵ^ while holding the minimum points fixed. Lower values result in more clusters forming as small clusters no longer merge, but result in more noise points. (b) Varying minimum points while holding ϵ^ fixed. The high ϵ^ minimizes noise points by merging clusters; small (minimum size 10) and large (minimum size 50) clusters are merged, while the medium cluster (minimum size 25) is stable and does not merge. (PNG) [file pgen.1012068.s003.png]

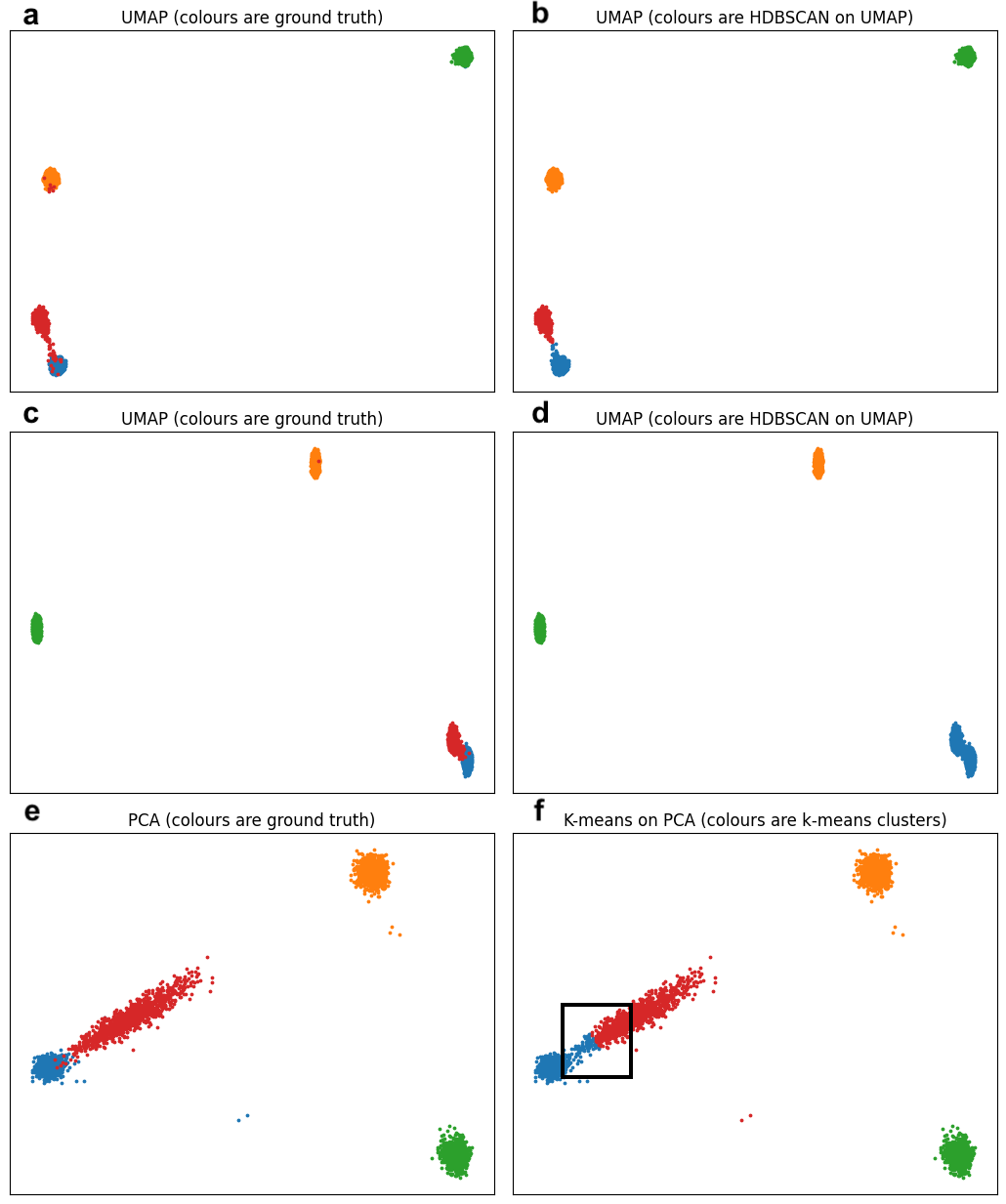

Supplement: S3 Fig — Simulated data of two source populations, an admixed population (with 25%/75% ancestry from the sources) and a fourth population. (a) UMAP of a replicate coloured by population label (considered ground truth here, though different ground truths could be considered). (b) The replicate from (a) coloured by HDBSCAN(ϵ^) returns four populations, reflecting discrete structure. (c) A different replicate, coloured by ground truth. (d) The replicate coloured by HDBSCAN(ϵ^); this time the algorithm merges two clusters. (e) PCA of the data, coloured by ground truth. (f) PCA, coloured by k-means (K=4). The box highlights how k-means groups part of the admixed population with a source population. (PNG) [file pgen.1012068.s004.png]

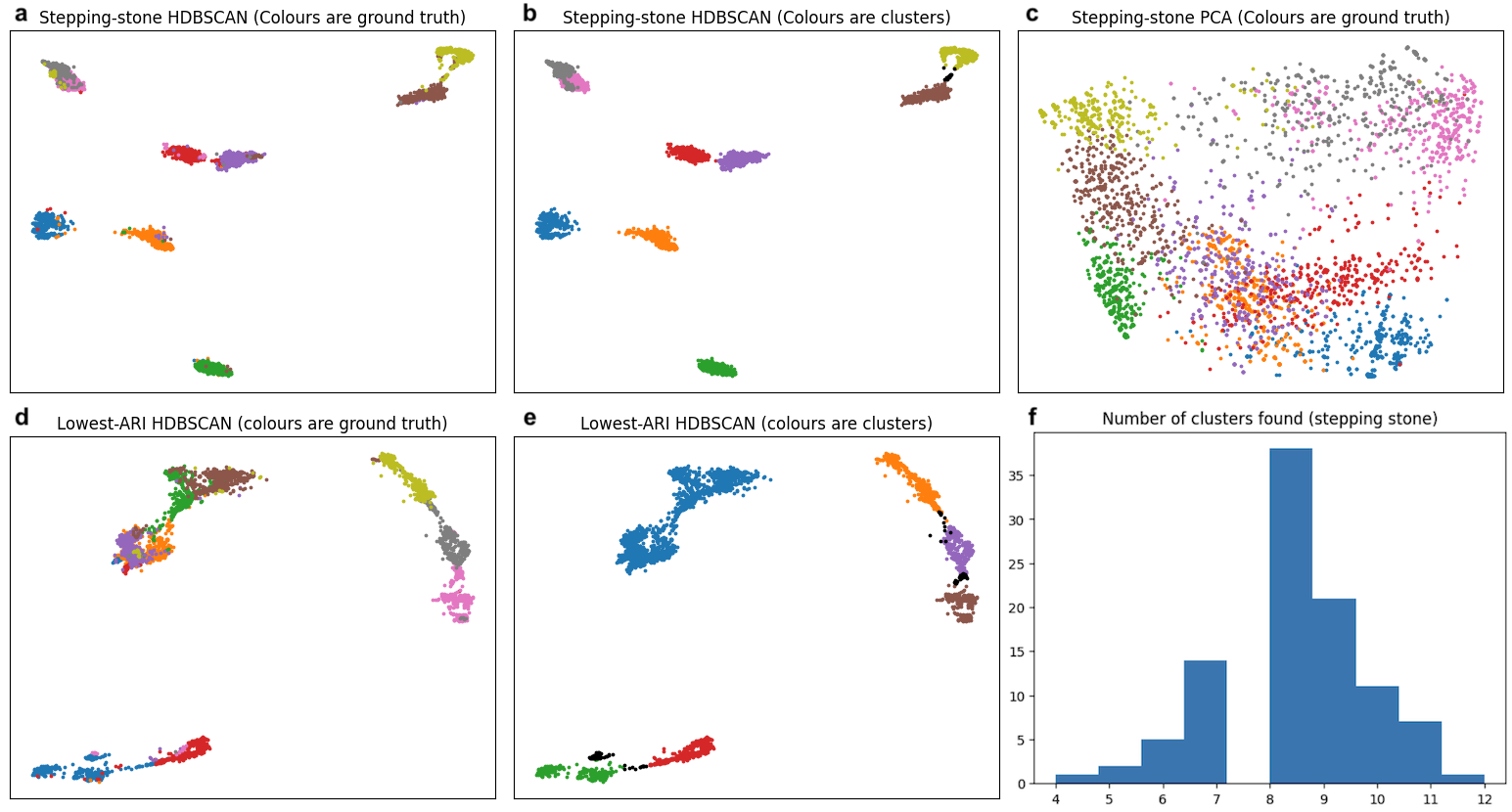

Supplement: S4 Fig — Simulated data of a stepping-stone model with nine populations of 500 with continuous migration. (a) UMAP coloured by population label (considered ground truth here, though different ground truths could be considered) (b) UMAP coloured by HDBSCAN(ϵ^) clusters. (c) PCA coloured by ground truth. In this data, population structure manifests as both discrete (from the stepping-stones) and continuous (from migration). (d) The replicate with the lowest ARI when comparing ground truth to HDBSCAN(ϵ^) coloured by ground truth. (e) The replicate from (d) coloured by HDBSCAN(ϵ^). Here the algorithm identifies some of the discrete structure, though it links many of the populations together from their migration. (f) The number of clusters identified across replicates. (PNG) [file pgen.1012068.s005.png]

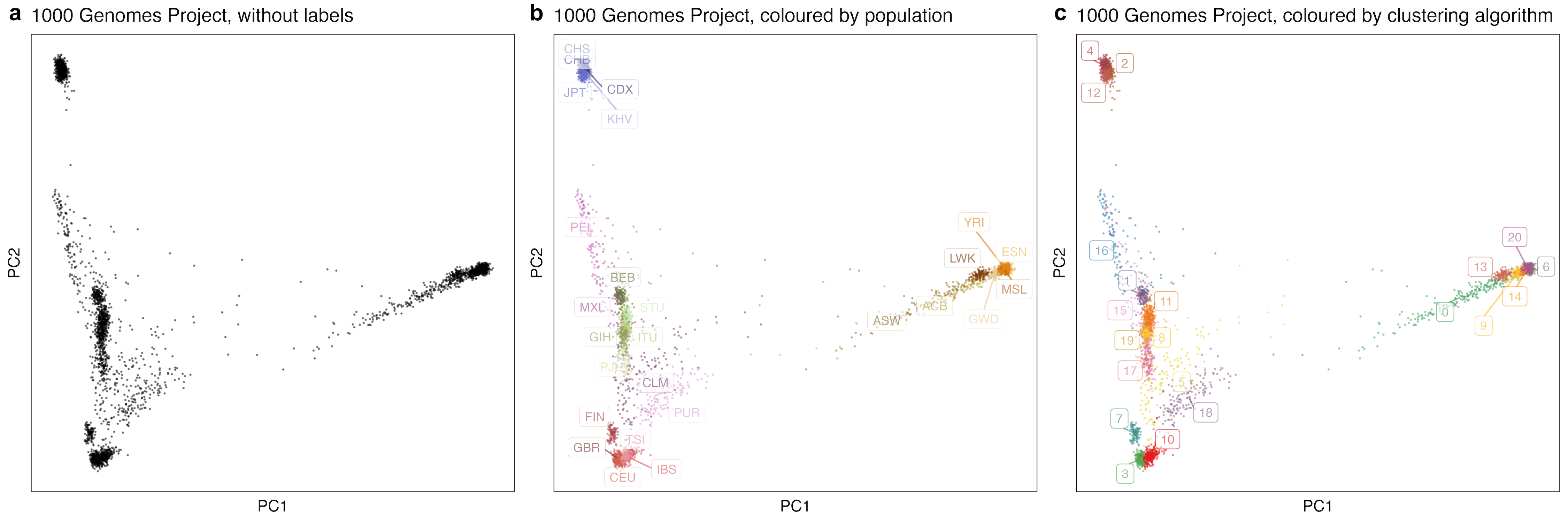

Supplement: S5 Fig — (a) No colours, to simulate unknown populations. (b) Coloured by population label. (c) Coloured by clusters derived from HDBSCAN(ϵ^). These are the same as in Fig 4c. (PNG) [file pgen.1012068.s006.png]

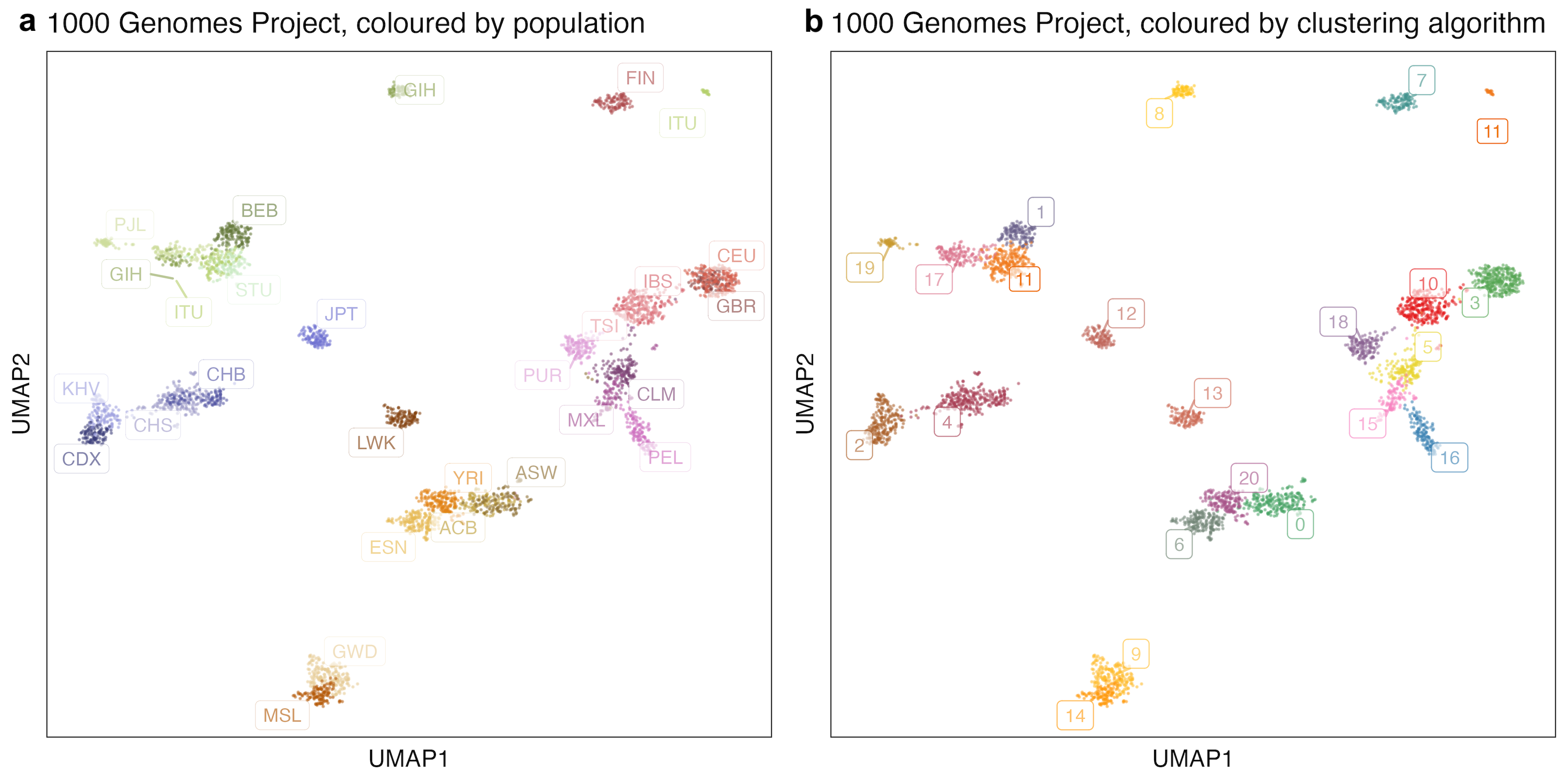

Supplement: S6 Fig — The UMAP used for visualization in Fig 4 was set to 50 neighbours. In this figure, we use 15 neighbours for the UMAP visualization, resulting in smaller visual clusters; the HDBSCAN(ϵ^) parameters are unchanged from the main text. (PNG) [file pgen.1012068.s007.png]

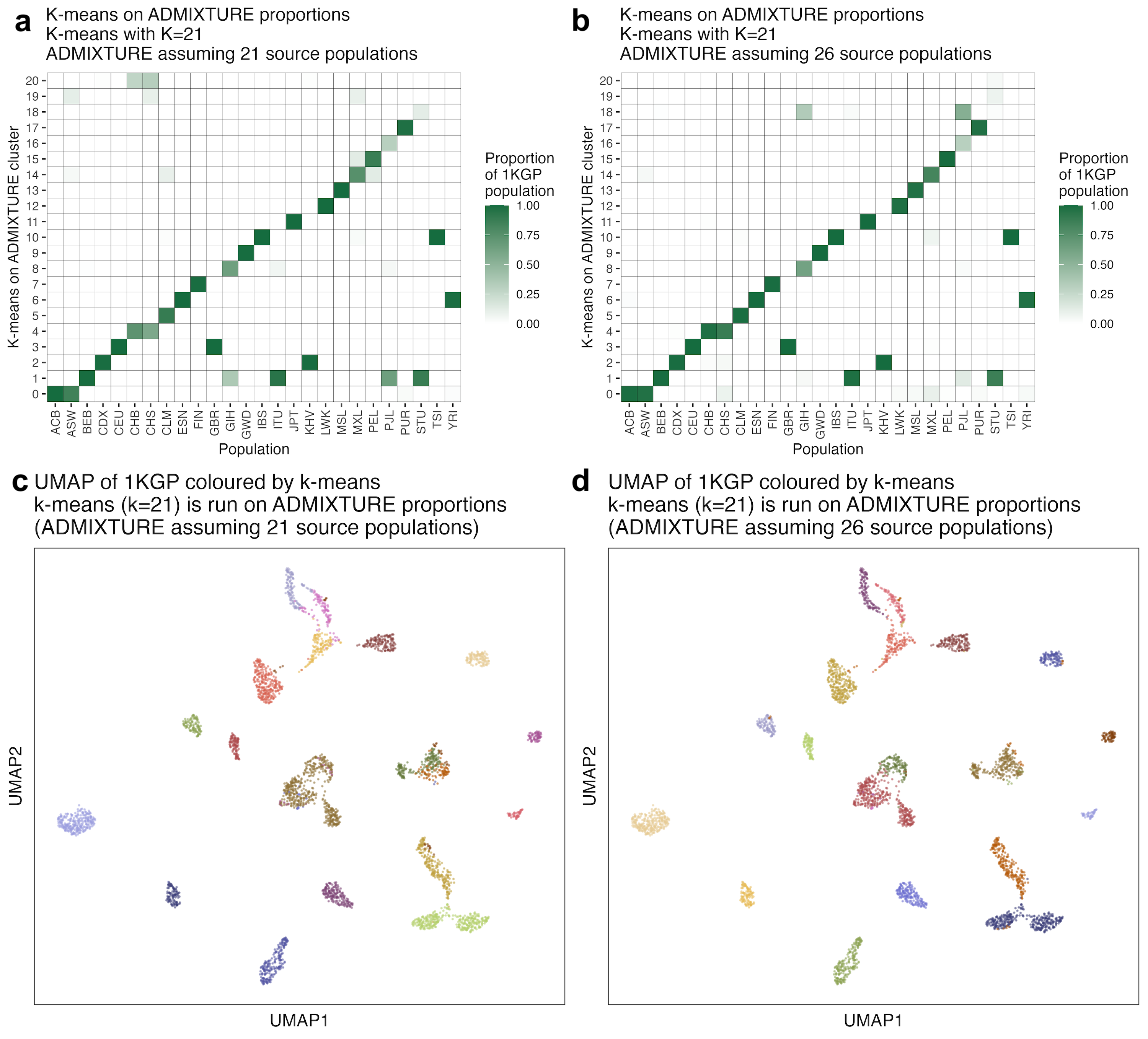

Supplement: S7 Fig — (a) Generating 21 clusters using k-means clustering on admixture proportions (K=21 populations specified). (PNG) [file pgen.1012068.s008.png]

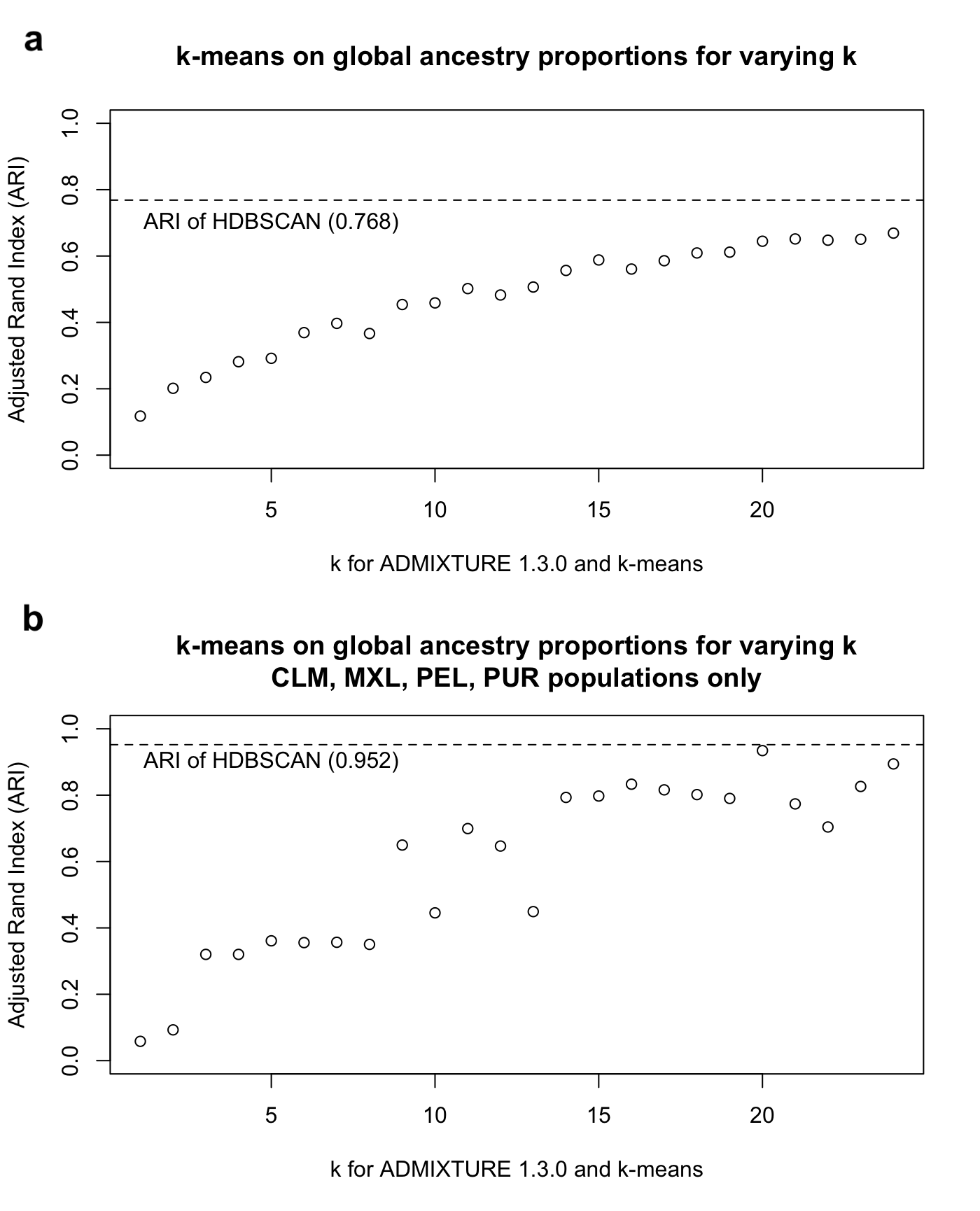

Supplement: S8 Fig — (PNG) [file pgen.1012068.s009.png]

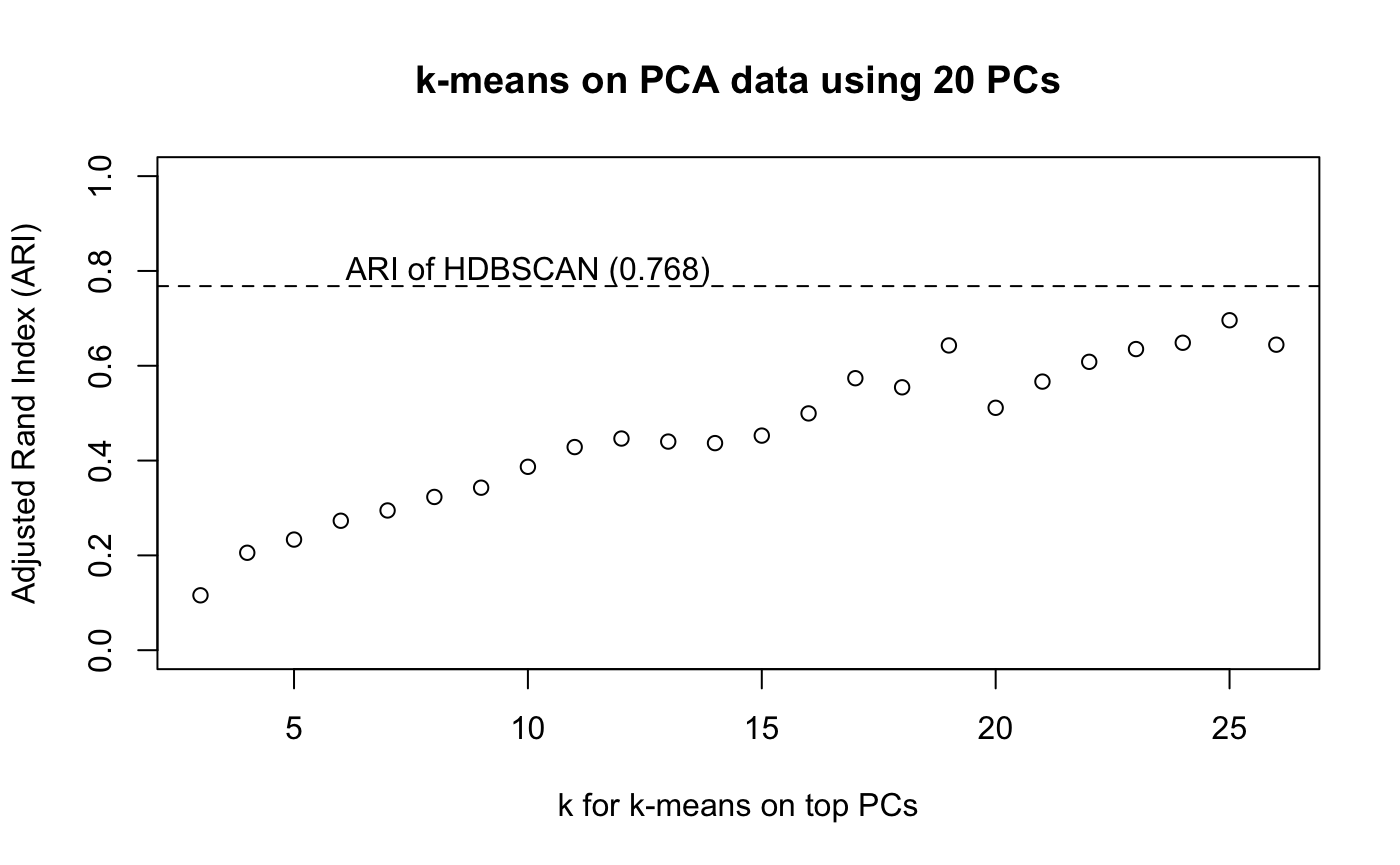

Supplement: S9 Fig — (PNG) [file pgen.1012068.s010.png]

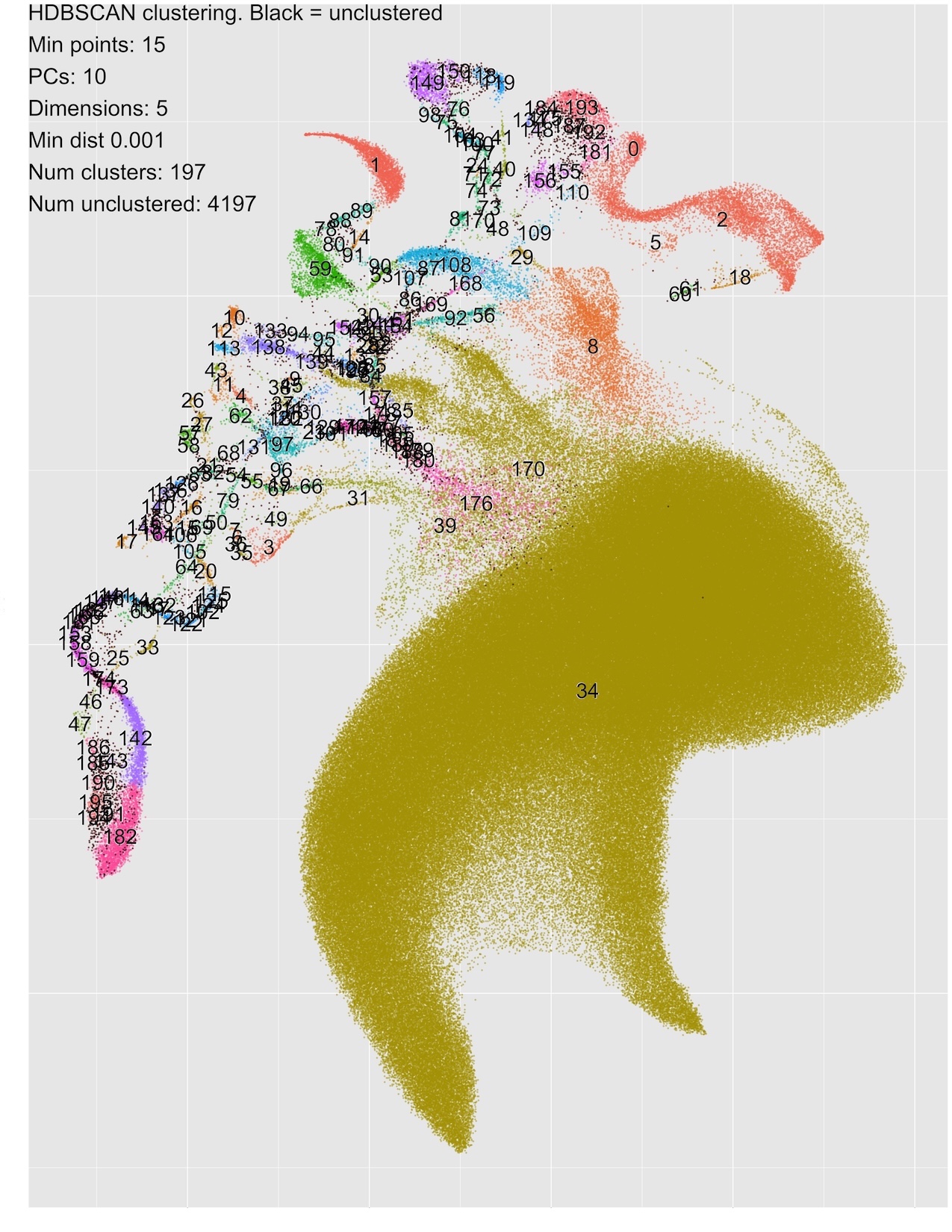

Supplement: S10 Fig — The algorithm fails to cluster many of the sub-populations, categorizing 4,197 individuals as noise and generated almost 200 micro-clusters. (PNG) [file pgen.1012068.s011.png]

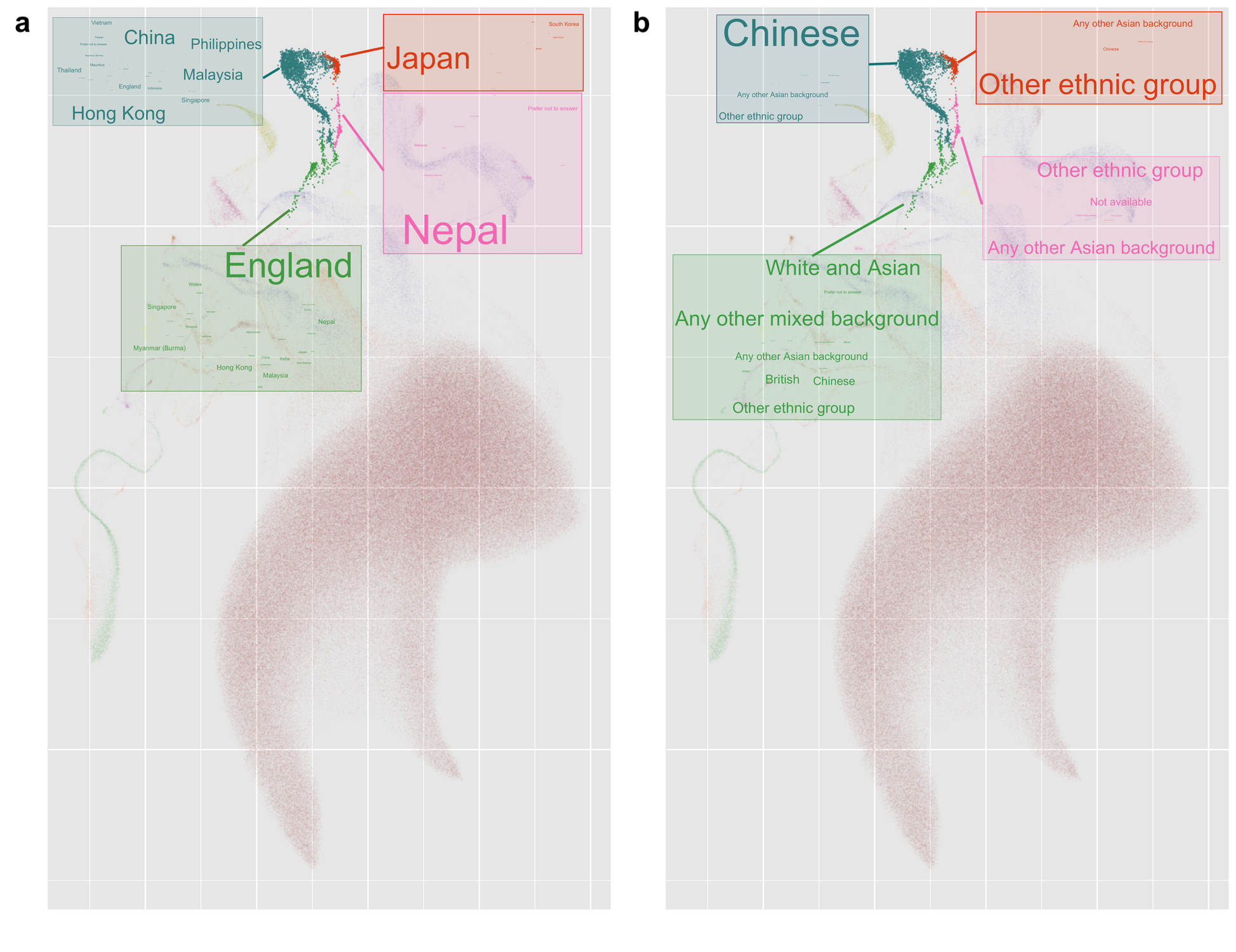

Supplement: S11 Fig — (a) Left: Word clouds of the most common countries of birth within each cluster. Most individuals in the orange cluster (Cluster 0) were born in Japan, and most in the pink cluster (Cluster 15) were born in Nepal. (b) Right: Word clouds for the most common EB. The most common in the blue cluster (Cluster 13) was “Chinese”, while those in the green cluster (Cluster 14) select a variety, including “White British”, “Chinese”, “Mixed”, or “Other”. Detailed breakdowns are available in S7 and S8 Tables. (PNG) [file pgen.1012068.s012.png]

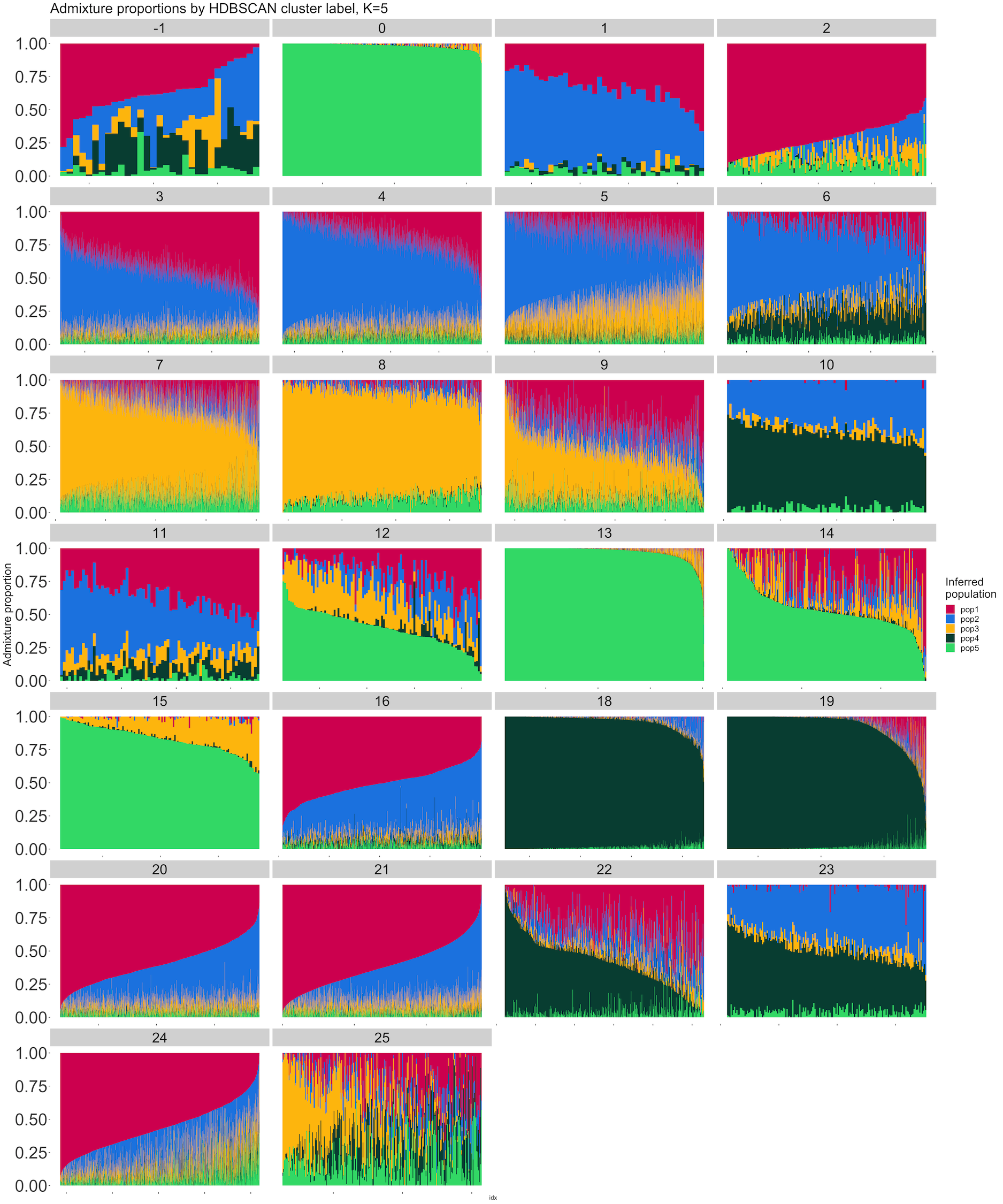

Supplement: S12 Fig — Cluster 17 (n>400,000) was excluded for computational reasons. Individuals not assigned to a cluster are labelled as −1. (PNG) [file pgen.1012068.s013.png]

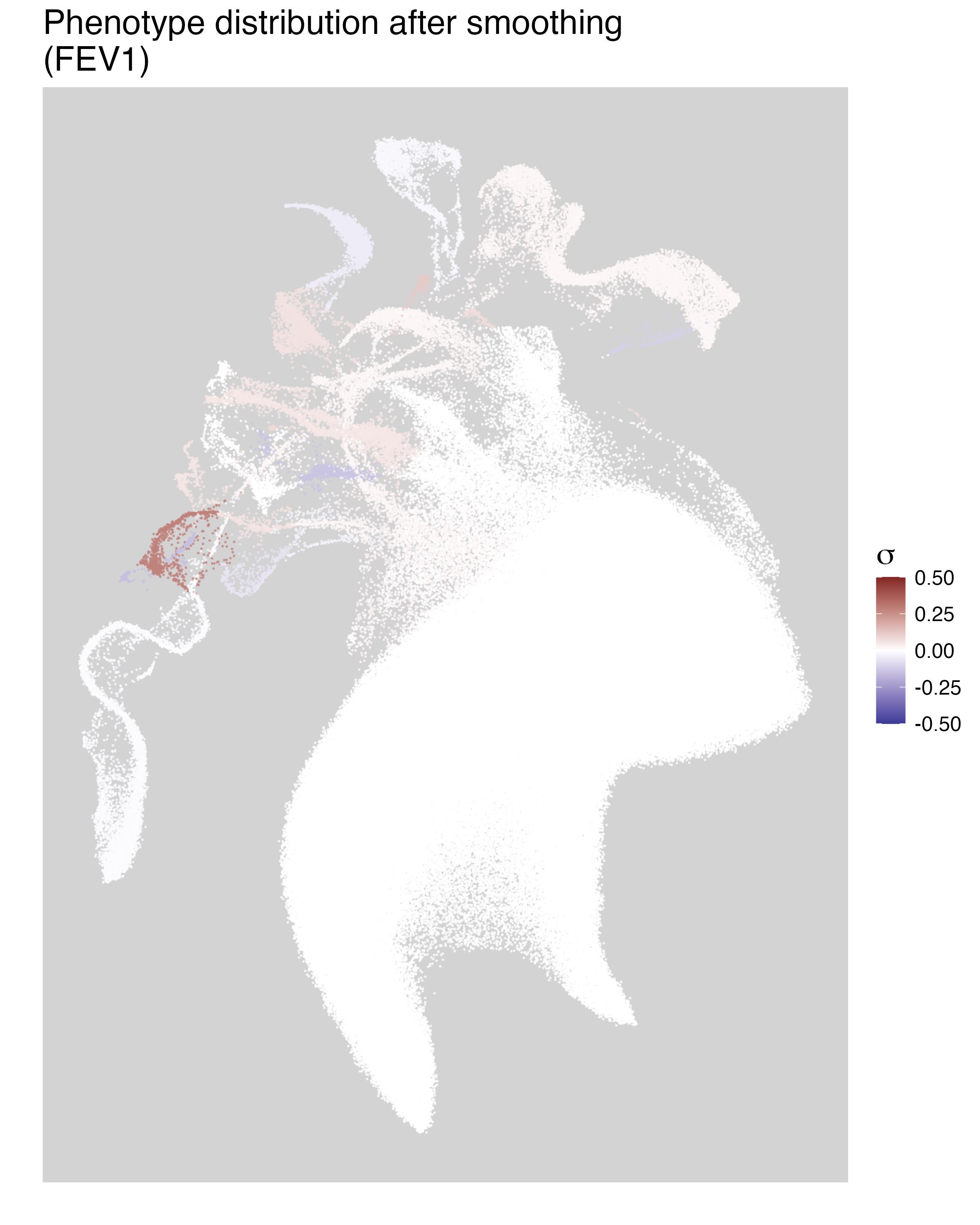

Supplement: S13 Fig — (JPEG) [file pgen.1012068.s014.jpeg]

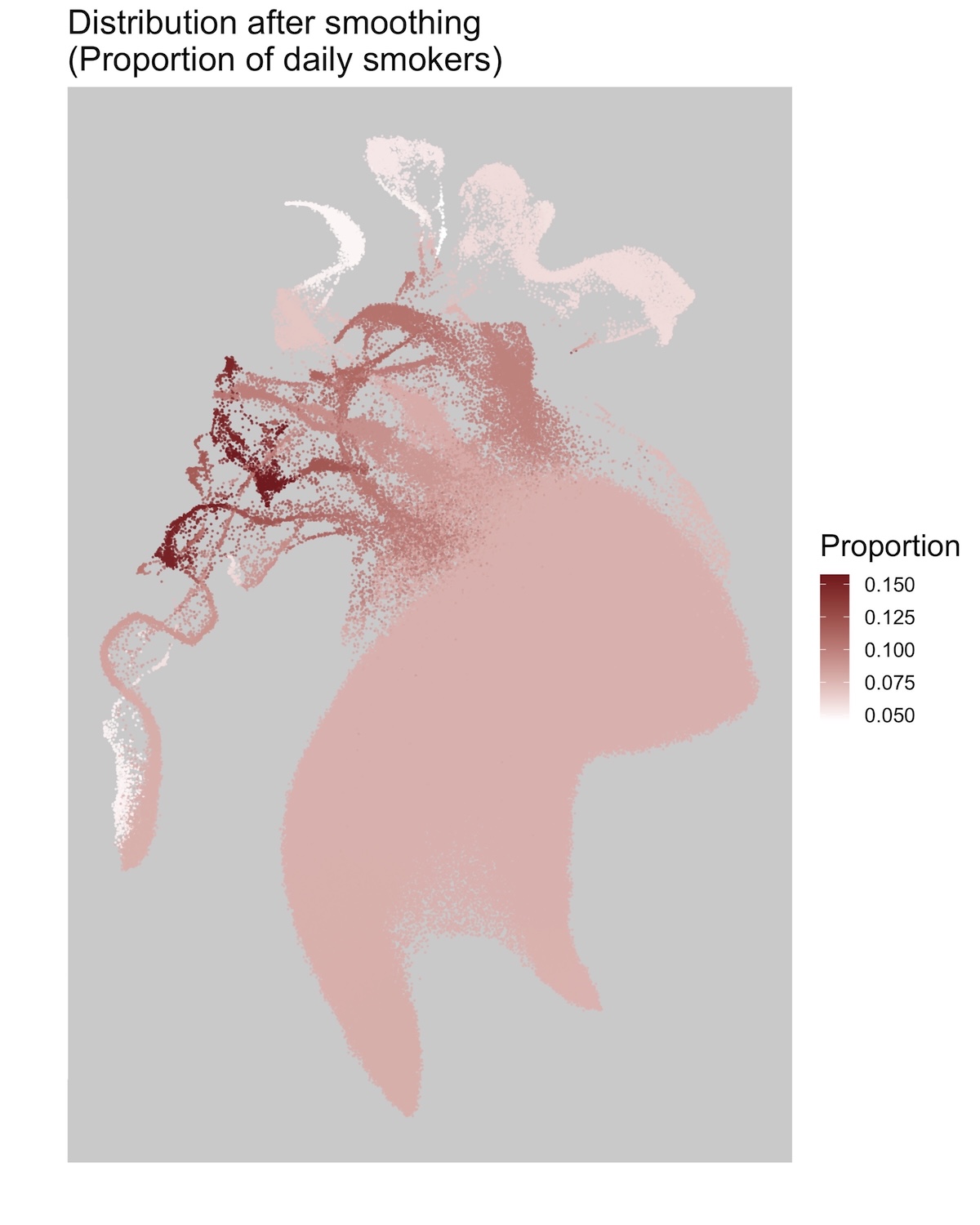

Supplement: S14 Fig — (JPEG) [file pgen.1012068.s015.jpeg]

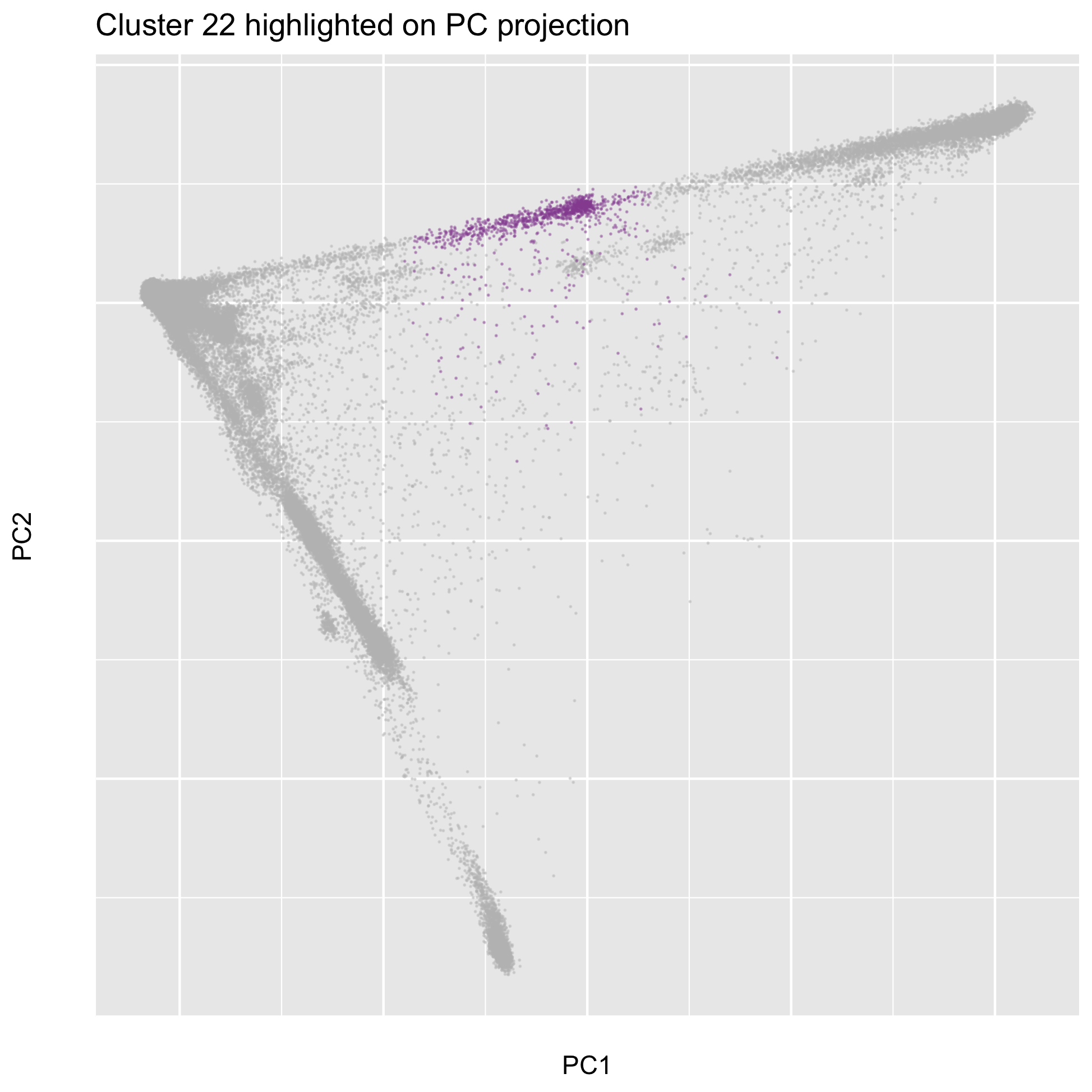

Supplement: S15 Fig — (PNG) [file pgen.1012068.s016.png]

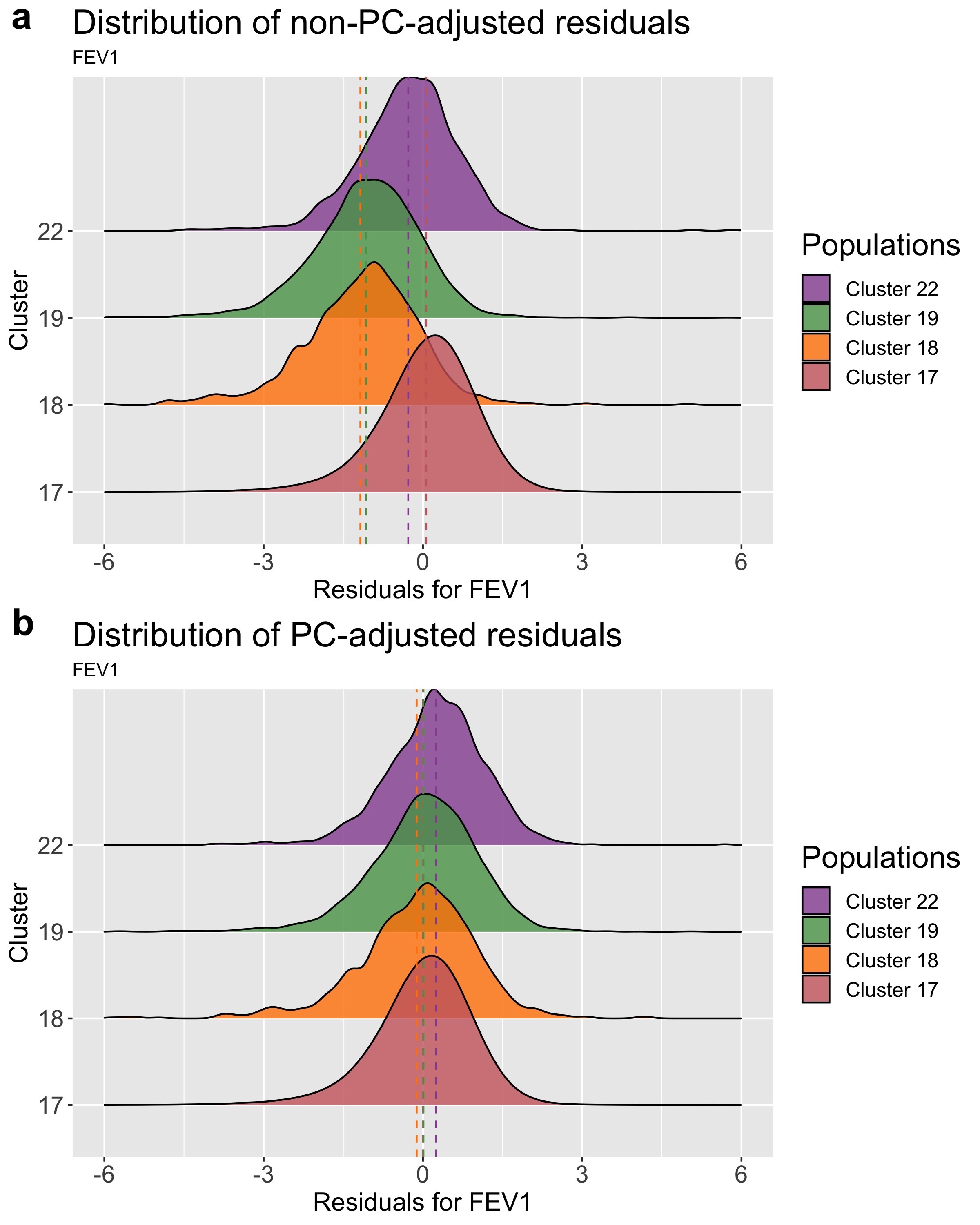

Supplement: S16 Fig — Vertical dotted lines represent the mean of the distribution. Cluster labels and colours match those in Fig 6a. Cluster 17 is mostly European-born individuals; Cluster 18 is mostly individuals born in African countries at or south of the equator; Cluster 19 is mostly individuals born in England, the Caribbean, Ghana, and Nigeria; and Cluster 22 is mostly individuals born in England who chose the EB “White and Black Caribbean” or “White and Black African”. (a) Top: Distribution of FEV1 by cluster without adjusting for population structure. (b) Bottom: Distribution of FEV1 by cluster after having adjusted for the top 40 PCs. (JPEG) [file pgen.1012068.s017.jpeg]

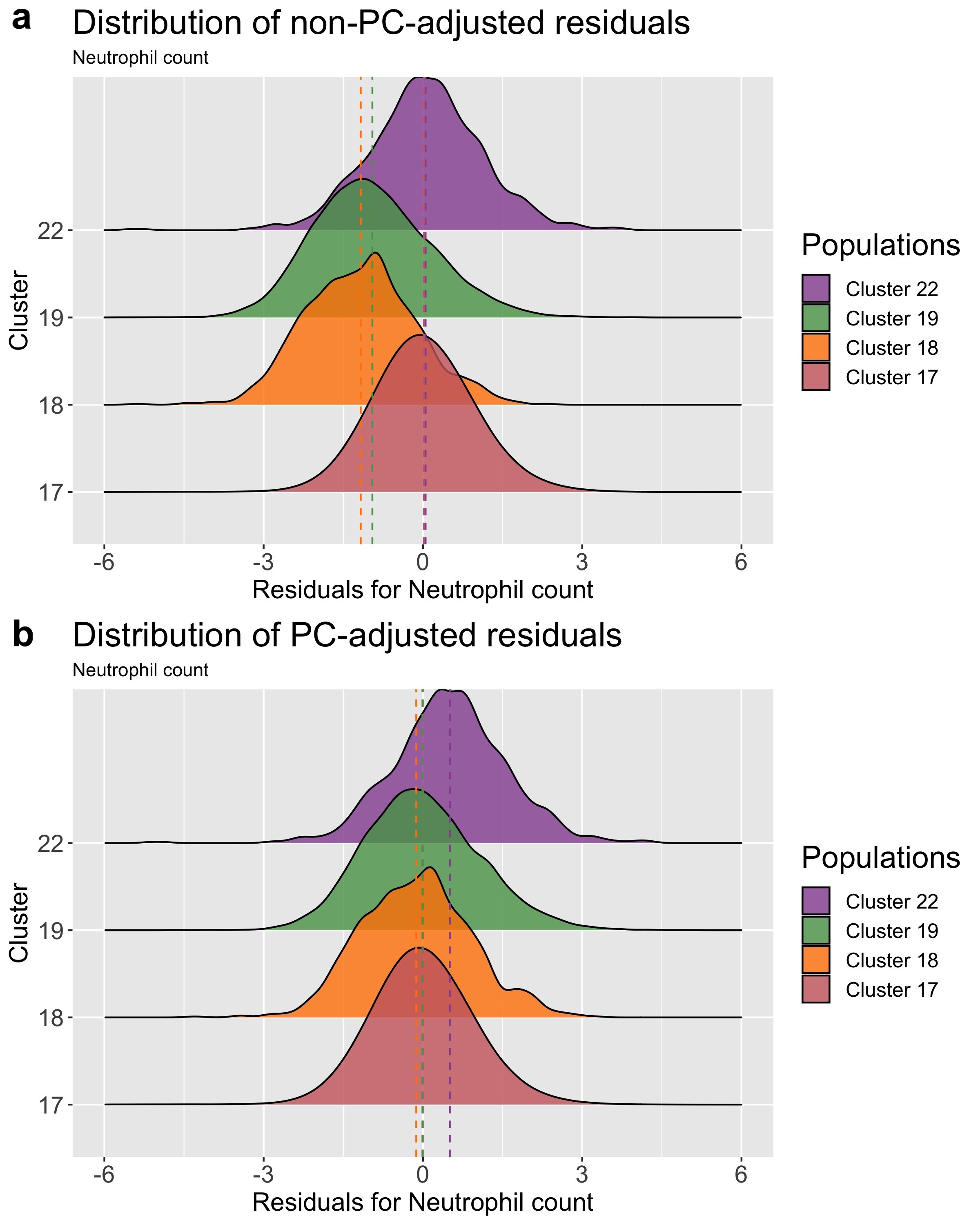

Supplement: S17 Fig — Vertical dotted lines represent the mean of the distribution. Cluster labels and colours match those in Fig 6a. Cluster 17 is mostly European-born individuals, Cluster 18 is mostly individuals born in African countries at or south of the equator, Cluster 19 is mostly individuals born in England, the Caribbean, as well as Ghana, and Nigeria in Western Africa, and Cluster 22 is mostly individuals born in England who chose the EB “White and Black Caribbean” or “White and Black African”. (a) Top: Distribution of neutrophil count by cluster without adjusting for population structure. (b) Bottom: Distribution of neutrophil count by cluster after having adjusted for the top 40 PCs. (JPEG) [file pgen.1012068.s018.jpeg]

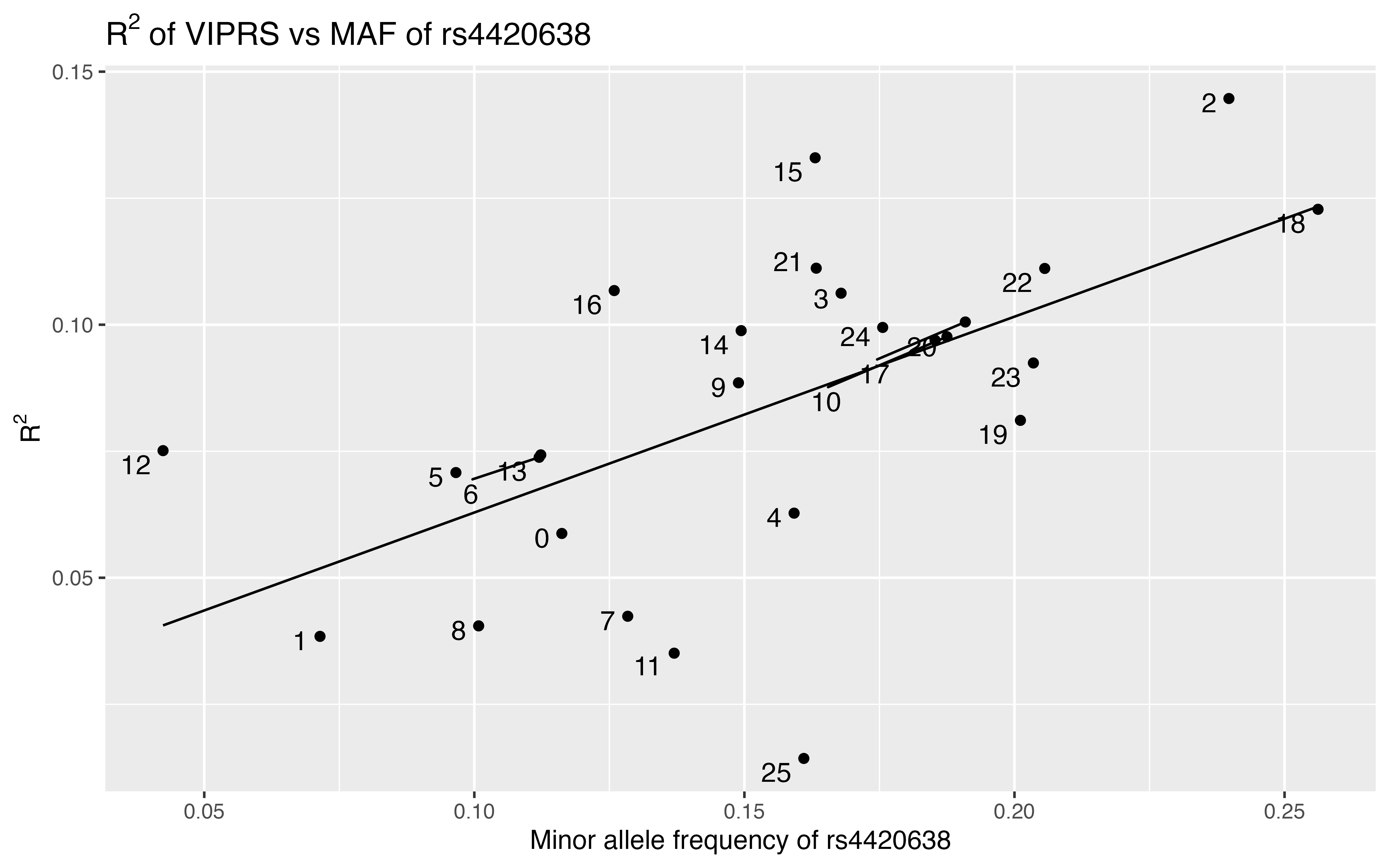

Supplement: S18 Fig — The regression summary is presented in S9 Table. (PNG) [file pgen.1012068.s019.png]

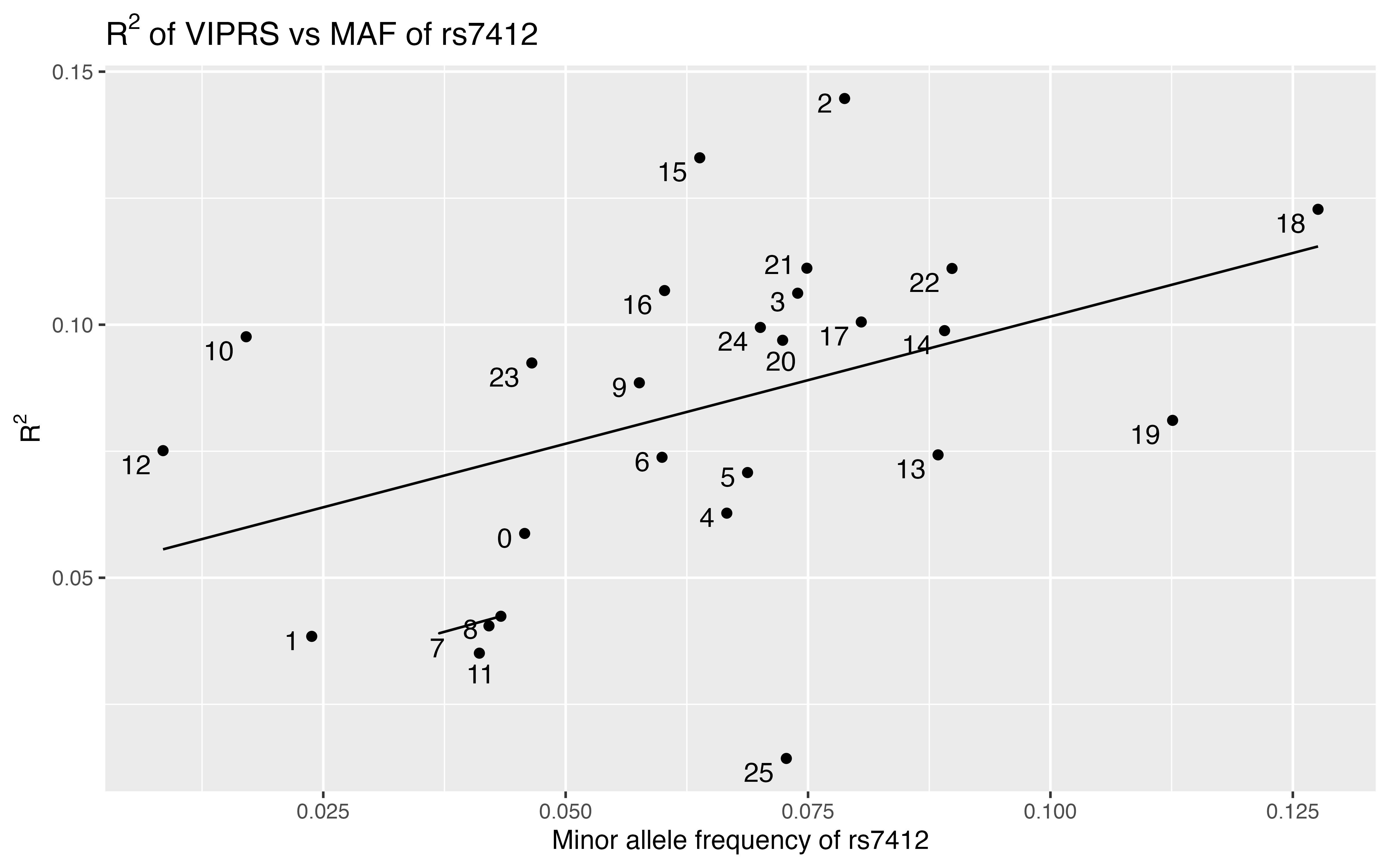

Supplement: S19 Fig — The regression summary is presented in S10 Table. (PNG) [file pgen.1012068.s020.png]

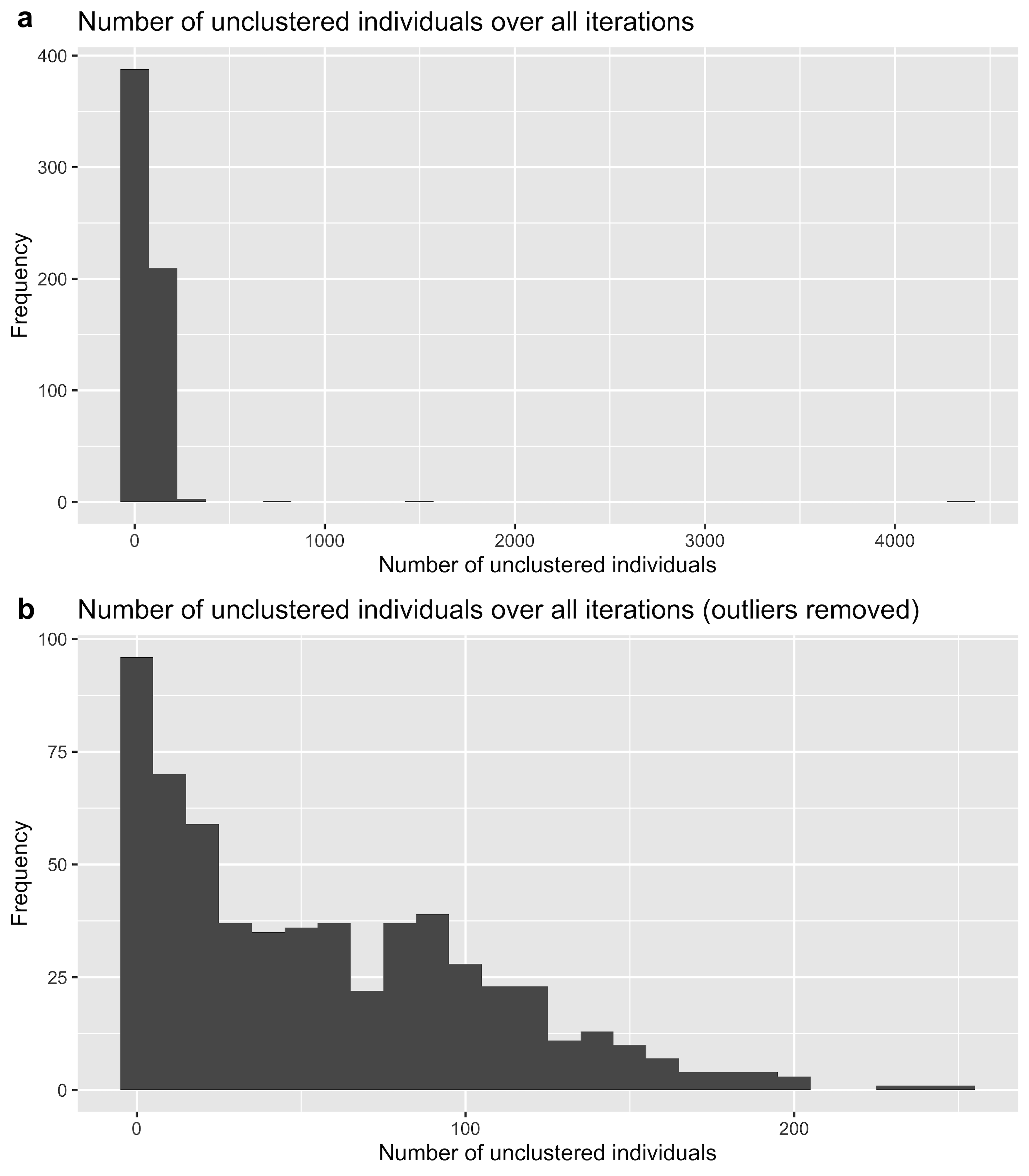

Supplement: S20 Fig — For 604 runs of UMAP-HDBSCAN(ϵ^) on the UKB, we count the number of individuals not assigned to a cluster. (a) Top: Across all 604 runs. (b) Bottom: To improve the scale of the figure, we remove 3 outlier runs in which 684, 1,535, and 4,346 individuals were not assigned to a cluster. (PNG) [file pgen.1012068.s021.png]

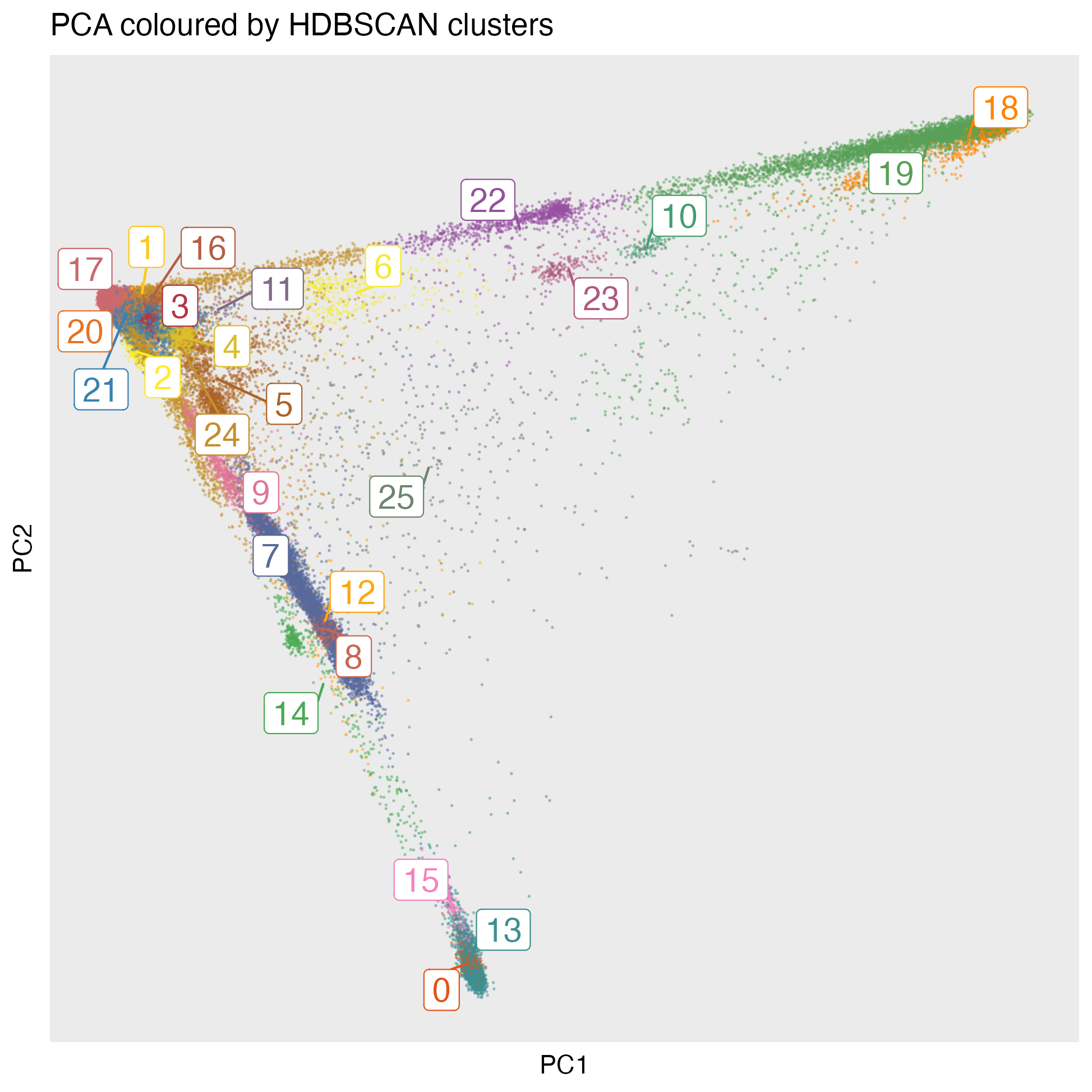

Supplement: S21 Fig — These are the same clusters used in Fig 6a. (PNG) [file pgen.1012068.s022.png]

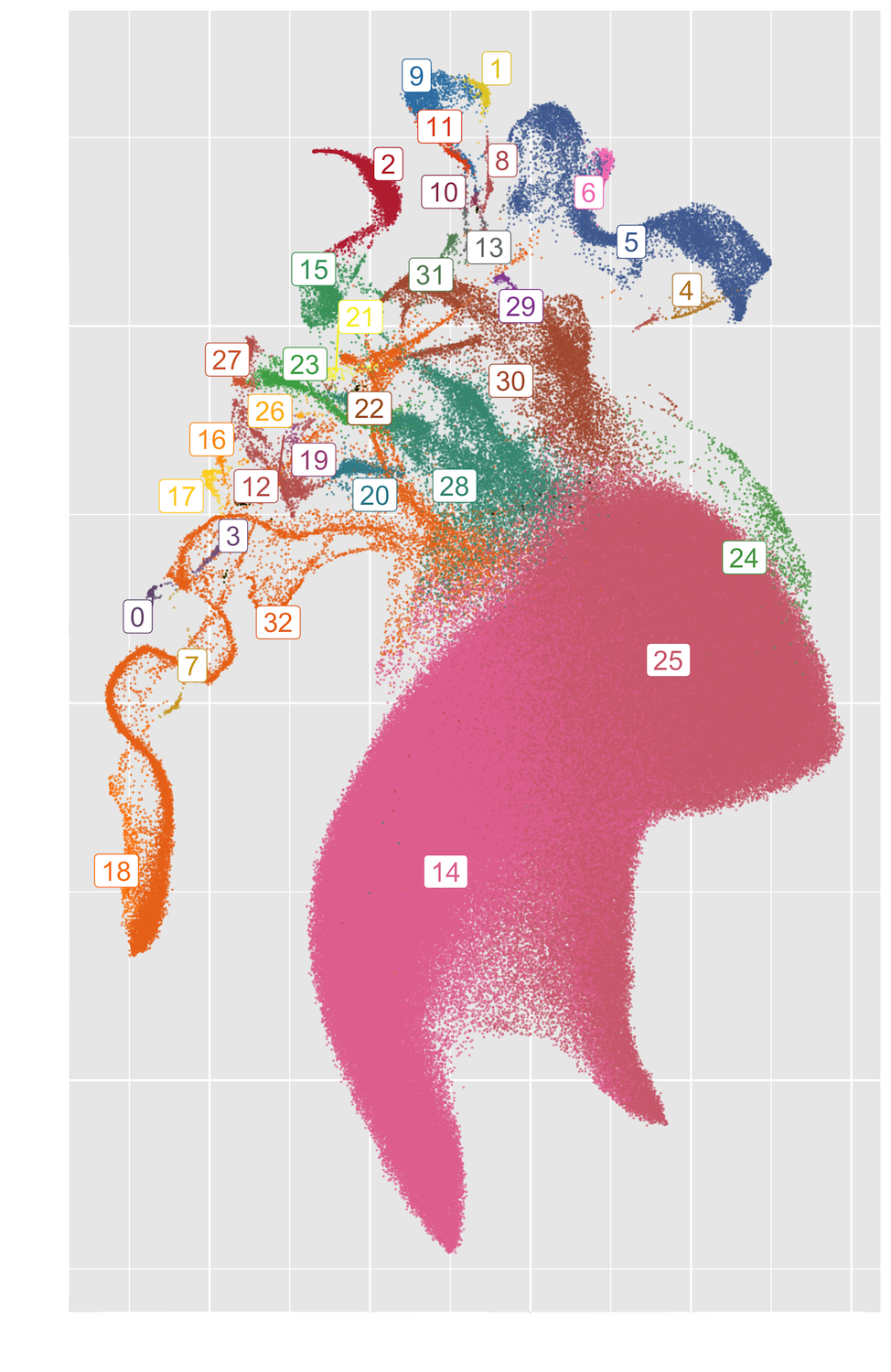

Supplement: S22 Fig — Compared to Fig 6a, the largest cluster (Cluster 17 in that figure) has been split into three smaller clusters (Clusters 14, 24, 25 in this figure). Other clusters have been split or merged, while some remain the same between runs. (PNG) [file pgen.1012068.s023.png]
